# Critical POU domain residues confer Oct4 uniqueness in somatic cell reprogramming

**DOI:** 10.1038/srep20818

**Published:** 2016-02-15

**Authors:** Wensong Jin, Lei Wang, Fei Zhu, Weiqi Tan, Wei Lin, Dahua Chen, Qinmiao Sun, Zongping Xia

**Affiliations:** 1 State Key Laboratory of Membrane Biology, Institute of Zoology, Chinese Academy of Sciences, Chaoyang, Beijing 100101, P.R.China; 2 State Key Laboratory of Stem Cell and Reproductive Biology, Institute of Zoology, Chinese Academy of Sciences, Chaoyang, Beijing 100101, P.R.China; 3 Life Sciences Institute and Innovation Center for Cell Signaling Network, Zhejiang University, Hangzhou, Zhejiang 310058, P.R.China

## Abstract

The POU domain transcription factor Oct4 plays critical roles in self-renewal and pluripotency of embryonic stem cells (ESCs). Together with Sox2, Klf4 and c-Myc, Oct4 can reprogram any other cell types to pluripotency, in which Oct4 is the only factor that cannot be functionally replaced by other POU family members. To investigate the determinant elements of Oct4 uniqueness, we performed Ala scan on all Ser, Thr, Tyr, Lys and Arg of murine Oct4 by testing their capability in somatic cell reprogramming. We uncovered a series of residues that are important for Oct4 functionality, in which almost all of these key residues are within the POU domains making direct interaction with DNA. The Oct4 N- and C-terminal transactivation domains (TADs) are not unique and could be replaced by the Yes-associated protein (YAP) TAD domain to support reprogramming. More importantly, we uncovered two important residues that confer Oct4 uniqueness in somatic cell reprogramming. Our systematic structure-function analyses bring novel mechanistic insight into the molecular basis of how critical residues function together to confer Oct4 uniqueness among POU family for somatic cell reprogramming.

The Oct4 protein of the POU (Pit1, Oct1/Oct2, UNC-86) family, together with Sox2 and Nanog, composes the core transcription factor circuitry that is essential for early embryogenesis and plays a central role in self-renewal and pluripotency of embryonic stem cells, as well as their differentiation into specific lineages^1^. It has also been well-documented that Oct4 functions in combination with Sox2, c-Myc and Klf4 (known as Yamanaka factors) to promote somatic cell reprogramming towards induced pluripotent stem cells (iPSCs), emphasizing the critical function of Oct4 in maintaining the ‘stemness’ of stem cells^2^,^3^.

Oct4 comprises three domains, a central POU domain flanked by an N-terminal and a C-terminal transactivation domain (TADs)^4^. The POU domain, composed of a specific domain (POU_S_), a POU homeodomain (POU_HD_), and a α-helix linker between the POU_S_ and POU_HD_ domains^5^, is responsible for specific binding to its target genes. The POU domain is highly conserved during evolution; but the N- and C-terminal TADs have been changed and exhibit little sequence conservation in the Oct4 family members^3^. Many studies have been focused on Oct4 functions regarding its interaction proteins, its target genes, its transcriptional regulation and its posttranslational modifications (PTMs) including phosphorylation^6^,^7^,^8^,^9^, O-glycosylation^10^, sumoylation^11^,^12^, and ubiquitination^13^,^14^, supporting a notion that posttranslational modifications serve as an important mechanism modulating Oct4 functions, and thus likely constitute a potential regulatory code in order to control the biological function of Oct4 in maintaining the self-renewal and pluripotency of stem cells, and their lineage specification as well.

Among the POU family members, only Oct4 plays pivotal roles in ES cell self-renewal and pluripotency^3^. Furthermore, Oct4 cannot be replaced by any other POU members in the induced pluripotent stem cell (iPSC) reprogramming assay^15^,^16^,^17^, suggesting that Oct4 is unique among the POU proteins. However, multiple sequence alignment of murine POU family members reveals there are no specific residues that are unique to Oct4, making it very intriguing in terms of the determining elements that make Oct4 unique. What are the specific residues or clusters that make Oct4 unique in the POU family? In other words, little is known about the molecular basis of the specific DNA binding sequences of Oct4 is determined. In addition, compared to the POU domain, much less attention has been on the function and regulation of the two Oct4 TAD domains^4^. Whether there are ES cell specific factors that specifically interact with the two TAD domains to regulate them thus controlling somatic cell reprogramming and the self-renewal and pluripotency of ES cells awaits investigation.

To understand the determinant elements of Oct4 uniqueness, in this study, we performed alanine scan on all the serine, threonine, tyrosine, lysine and arginine residues and putative DNA binding residues of murine Oct4. Our data suggest that the N- and C-terminal TAD domains of Oct4 are required but are not unique which could be functionally replaced by the TAD domain from YAP for somatic cell reprogramming. Notably, we uncovered a series of residues that are important for Oct4 functionality, in which almost all of these key residues are located within the POU domain of Oct4, suggesting that the POU domain is critical for Oct4 function. Moreover, we uncovered two important residues that confer Oct4 uniqueness in somatic cell reprogramming. Collectively, our systematic structure-function analyses bring novel mechanistic insights into molecular understanding of how critical residues function together to confer Oct4 DNA binding specificity and make it unique among POU family for somatic cell reprogramming.

## Results

### Generation of an Oct4 mutant library to identify functional residues for somatic cell reprogramming

In order to better understand the determinant elements of Oct4 uniqueness, in this study, we took an unbiased approach to investigate those residues that are potential PTM residues including all the serine, threonine, tyrosine, lysine and arginine residues (e.g. S, T, Y, K and R), and those putative DNA binding residues of murine Oct4 (mOct4). We changed them, either individually or in combination if the consecutive residues are identical, to alanine and tested their functions using somatic cell reprogramming assays. By this way, we generated a mOct4 mutant library containing 90 mOct4 mutants covering 95 sites across the full-length murine Oct4A sequence (Supplementary Table 1). All the mutants have been subjected to DNA sequencing to verify their correctness before their further functional tests.

**Figure 1:**
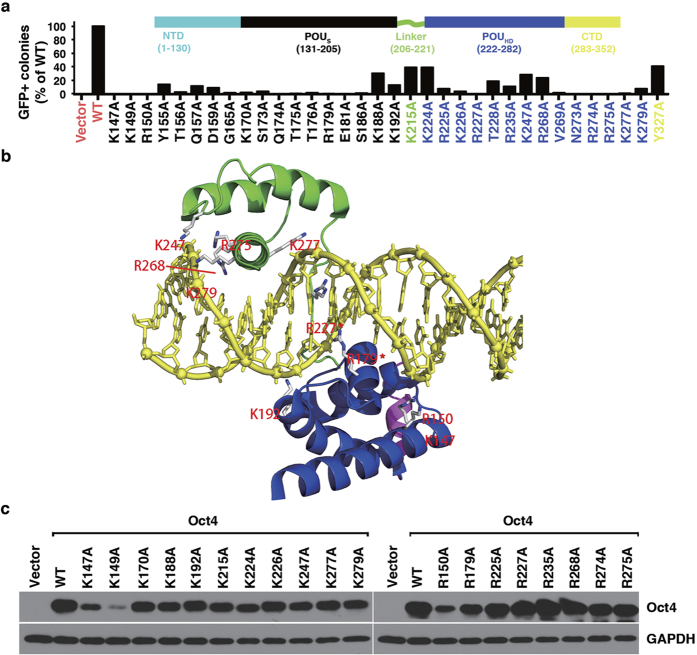
Identification of critical Oct4 residues for somatic cell reprogramming by alanine scanning. (**a**) Percentage of GFP+ iPSC formation by Oct4 alanine mutants, shown here are defective ones. For complete data see Supplementary Fig. S1c and Supplementary Table S1). (**b**) Modeling of Oct4^POU^:PORE complex. The POU_S_ domain is shown in blue, the POU_HD_ domain in green, the linker in magenta, and the DNA in yellow. K and R residues, except for K147 (4.4 Å), that have atoms within 3.6 Å of DNA (protein-DNA interface) are highlighted and those in contact with the DNA base are labeled with a star (*). The residue numbering in the model is in accord with positions in murine Oct4 (mOct4). This labeling and coloring is maintained throughout this manuscript. (**c**) Protein stability of reprogramming defective mOct4 K-to-A and R-to-A mutants in MEFs.

Oct4 has been shown to be essential for somatic cell reprogramming towards iPS cells. We therefore employed the somatic cell reprogramming assay to test whether the mutated sites were required for Oct4 to promote iPS cell formation by using combinations of Oct4, Sox2, Klf4, and c-Myc (OSKM, the Yamanaka factors)^2^. For some mutants, we also employed another commonly used reprogramming regime by using combinations of Oct4, Sox2, Klf4, and Nanog (OSKN)^18^. Using previously described methods of retrovirus transduction, we introduced the transcription factors Sox2, Klf4, and c-Myc (or Nanog), in combination with the Oct4 wild-type (WT) (OSKM, OSKN) or its mutated form (O^m^SKM, O^m^SKN) into murine embryonic fibroblasts (MEFs) derived from Oct4-green fluorescent protein (GFP) mice and measured the efficiency of the GFP-positive (GFP+) colony formation (Supplementary Fig. S1a)^19^. As shown in Supplementary Fig. S1b, there were absolutely no GFP+ colonies without Oct4 in the reprogramming regimes, a prerequisite needed for success of our assays to test our mutant library. Transduction of OSK gave rise to significant reprogramming efficiency. The efficiency was further enhanced by the inclusion of either c-Myc (OSKM) or Nanog (OSKN) in the reprogramming regime (Supplementary Fig. S1b). We then measured the efficiency of the GFP-positive colony formation for all the mOct4 mutants (Fig. 1a and Supplementary Fig. S1c). We found that, while 56 of 90 mutant forms of Oct4 in combination with Sox2, Klf4, and c-Myc produced a comparable number of GFP-positive colonies to wild-type Oct4, 34 mutants covering 34 sites, generated significantly fewer or no iPSC colonies (Supplementary Fig. S1c).

Notably, we found that, among the 34 mutants that showed defects in reprogramming, except Y327A mutant, which gave rise to about 45% colonies of WT, all the other 33 mutants harbor mutation sites within the POU domain of Oct4 (Fig. 1a). This result demonstrates that the activity of the Oct4 POU domain is extremely critical for Oct4 functionality in reprogramming somatic cells into iPS cells. This result also suggests that those putative PTM residues, if there are any, at the N- and C-termini outside of the Oct4 POU domain might not be essential for regulation of Oct4 function, at least during the reprogramming process (see more below).

### Reprogramming defective K and R mutants contain residues involved in direct DNA binding

Close examination of the recently reported co-crystal structure of mOct4/DNA complex (PDB: 3L1P)^5^ revealed that most of the lysine (K) and arginine (R) residues with dramatic defects in the reprogramming assays are involved in direct interaction with DNA backbones or bases through formation of hydrogen bonds. These residues include K147, R150, R179, K192, R227, K247, R268, R275, K277, and K279 (Fig. 1b, Supplementary Fig. S1f and Supplementary Table 1). Our findings revealed that mutating these Lys and Arg residues to alanine interferes with Oct4-DNA interactions, consequently influencing the induction of downstream pluripotency genes and the reprogramming of somatic cells into iPSCs (Supplementary Fig. S1g). In agreement with the structural analysis, multiple sequence alignment shows that most of the defective Oct4 lysine and arginine residues are highly evolutionarily conserved (Supplementary Fig. S1d).

We further characterized these mutants in terms of their protein stability, subcellular localization, and transactivation activities using 6xCR4-Luc^20^ and 5xW-Luc reporter assays^21^ (Supplementary Fig. S1j, S1k and S1l). All the mutants showed mainly nuclear localization without any detectable defects (Supplementary Fig. S1m). In addition, except K147A, K149A, and R150A mutants, the other K-to-A and R-to-A mutants showed protein expression levels comparable to Oct4 WT (Fig. 1c). Accordingly, it is also these three mutants that showed dramatic defects in the reporter assays (Supplementary Fig. S1h and S1i). These three residues locate at the end of the first α-helix of the POUs subdomain, from where it starts to interact with DNA. Instability of these three mutant proteins after transduction into MEF cells suggests that this positive charged motif is very important for regulating Oct4 stability, which also implies that DNA binding of Oct4 might play a critical role in stabilizing Oct4 protein.

### Reprogramming defective S/T/Y mutants contain residues involved in DNA binding

**Figure 2:**
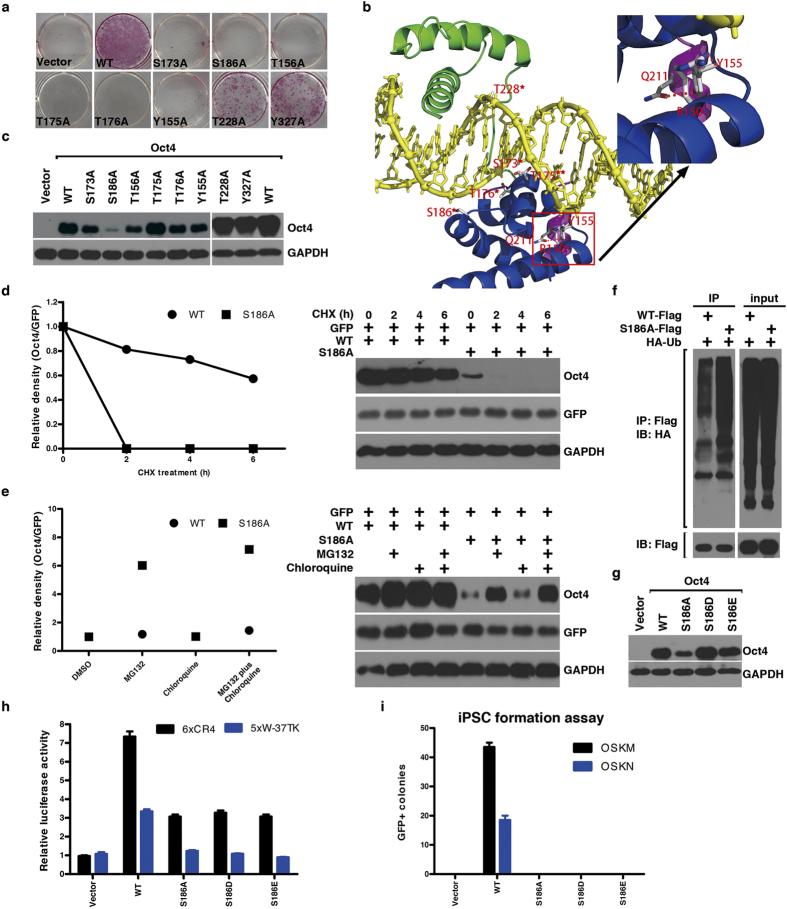
Characterization of reprogramming defective mOct4 S-to-A, T-to-A and Y-to-A mutants. (**a**) AP staining of reprogramming defective S-to-A, T-to-A and Y-to-A mutants. (**b**) Model of Oct4^POU^:PORE complex. The POU_S_ domain is shown in blue, the POU_HD_ domain in green, the linker in magenta, and the DNA in yellow. Ser/Thr/Tyr residues that have atoms within 3.6 Å of DNA (protein-DNA interface) are highlighted and those in contact with the DNA base or phosphates are labeled with two stars or one star, respectively. R150, Y155 and Q211 are highlighted to show their intra-molecular interactions, and hydrogen bonds are represented by red dot lines. Right upper panel shows zoomed-in view of the intra-molecular interactions. (**c**) Protein stability of reprogramming defective S-to-A, T-to-A and Y-to-A mutants in MEFs. (**d**) CHX-based assay was used to determine the protein turnover of S186A, as compared to WT, in HEK293 cells. The corresponding immunoblotting is at the right. (**e**) MG132 and chloroquine-based assays were used to determine the main means by which S186A was degraded. The corresponding immunoblotting is at the right. (**f**) Enhanced polyubiquitination of S186A mutant compared to WT. (**g**) Comparison of protein stability of S186A, S186D and S186E mutants in MEFs. (**h**) The relative transcriptional activity of S186A, S186D and S186E mutants was measured using 6xCR4-Luc and 5xW-Luc reporters, respectively. Data are representative of at least three independent experiments. Statistics analysis was performed using a t-test (mean and s.d. of duplicate assays). (**i**) Generation of GFP + iPSC colonies by the S186A, S186D and S186E mutants. Data are representative of three independent experiments. Statistics analysis was performed using a t-test (mean and s.d. of duplicate assays).

We next focused our attention on Ser, Thr and Tyr residues, which are potential phosphorylation sites. After changing all the Ser, Thr and Tyr residues of mOct4 to Ala, we found, except for Y155, T156, S173, T175, T176, S186, T228 and Y327 (Fig. 2a), all the other mutants were functionally indistinguishable from Oct4 WT (Supplementary Fig. S1c). It is interesting to point out that Y155, T156, S173, T175, T176, S186 and T228 are well within the POU domain. Sequence alignment reveals that these sites are highly conserved in the POU family from different species (Supplementary Fig. S1d). Sequence alignment also reveals that T156, S173, T175, T176, S186 and T228 are also highly conserved in the murine POU domain-containing family members (Supplementary Fig. S1e). The evolutionary conservation of these sites suggests their functional importance in the iPSC reprogramming and self-renewal and pluripotency of ES cells. Detailed analysis of the Oct4 POU domain complexed to DNA indicates that S173, T176, S186 and T228 all interact with the DNA backbone and T175 contacts base directly (Fig. 2b and Supplementary Fig. S2a). Thus, mutating them to Ala might affect their interaction with DNA and target gene induction in the iPSC process, which is consistent with our reporter assays (Supplementary Fig. S2b and S2c).

Protein stability testing shows that S186A had much reduced expression level (Fig. 2c). CHX-based analysis revealed the S186A mutant has much shorter half-life than WT, which undergoes ubiquitin-proteasome-dependent turnover, as treatment with proteasome inhibitor MG132, but not lysosome inhibitor chloroquine, dramatically enhanced its detectable protein level (Fig. 2d–f).

To further characterize S186 and test possible phosphorylation in its regulation, we mutated it to aspartic acid (D) or glutamic acid (E) to mimic phosphorylation. After changing to D or E, the protein expression level was brought up to that of WT (Fig. 2g), but the transactivation activity was further reduced in the reporter assays (Fig. 2h). In the iPSC assays, the Oct4 S186D or S186E failed to rescue its activity (Fig. 2i). These results suggest phosphorylation of S186 would regulate Oct4 protein stability but might negatively regulate its transactivation activity. We would argue that free Oct4 should be phosphorylated at S186 to prevent it from degradation and that cycling of S186 between phosphorylation and dephosphorylation regulates Oct4 protein stability and its DNA binding.

### Critical POU domain residues in differential induction of Oct4 target genes

**Figure 3:**
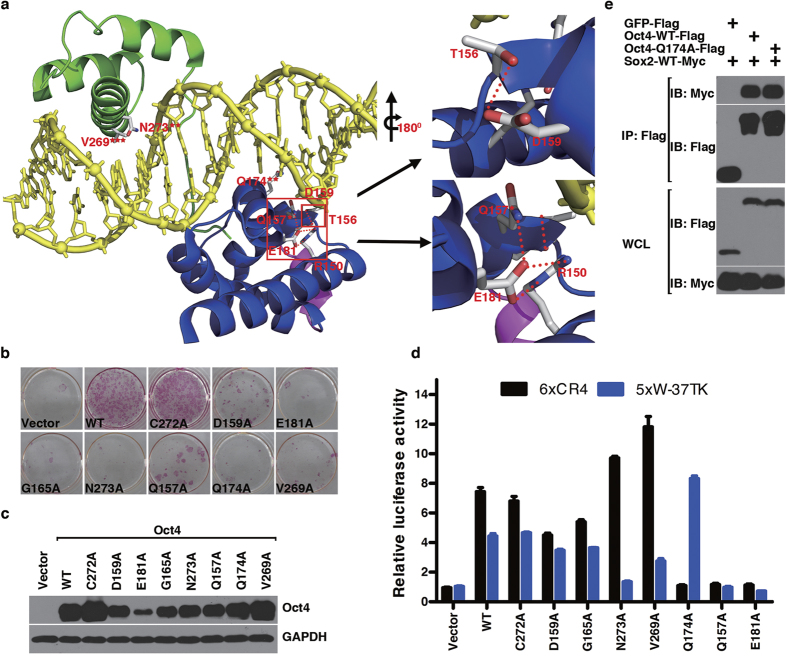
Characterization of mOct4 critical DNA binding residues. (**a**) Model of Oct4^POU^:PORE complex. The POU_S_ domain is shown in blue, the POU_HD_ domain in green, the linker in magenta, and the DNA in yellow. Residues tested in our collection that have atoms within 3.6 Å of DNA (protein-DNA interface) are highlighted. Those making hydrogen bonds to phosphate or DNA bases are labeled with one star or two stars, respectively. V269 is labeled with three stars for its non-polar contacts with DNA base. R150, T156, D159 and E181 are highlighted to show their intra-molecular interactions. Right upper panel shows zoomed-in view of the interaction between D159 and T156. Right lower panel shows zoomed-in view of the interaction among E181, R150 and Q157. Hydrogen bonds are shown by red dot lines. (**b**) AP staining of reprogramming defective alanine mutants. (**c**) Protein stability of reprogramming defective alanine mutants in MEFs. (**d**) The relative transcriptional activity of reprogramming defective alanine mutants was measured using 6xCR4-Luc and 5xW-Luc reporters, respectively. Data are representative of at least three independent experiments. Statistics analysis was performed using a t-test (mean and s.d. of duplicate assays). (**e**) Interaction of mOct4 Q174A with Sox2 was compared to that of Oct4 WT with Sox2.

Encouraged by the findings with S/T/Y/K/R, we characterized some other non-S/T/Y/K/R residues that make contacts with DNA base or backbone phosphate, or form hydrogen bonds with other functionally important Oct4 residues based on the crystal structure of mOct4-DNA complex (Q157, D159, G165, Q174, E181, V269, C272 and N273) (Fig. 3a and Supplementary Fig. S3a). To test this, we mutated them to Ala individually and tested them in the reprogramming assay. As shown in Fig. 3b, except for C272A mutant, which showed no defect in reprogramming efficiency, the other 7 showed very strong defects, among which Q174A, E181A, V269A and N273A mutants didn’t produce any single GFP+ colonies. Protein stability and subcellular localization tests showed that, except E181A mutant showing reduced protein expression level, all the other mutants didn’t show much difference from mOct4 WT (Fig. 3c and Supplementary Fig. S3b). Although the 7 mutants all showed defects in reprogramming, they displayed compound activities in the reporter assays. As shown in Fig. 3d, on one hand, D159A and G165A didn’t have significant defects in both the 6xCR4-Luc and the 5xW-Luc reporter assays, whereas Q157A and E181A displayed almost no activities. On the other hand, V269A and N273A had strong activities towards the 6xCR4 reporter but weak activities towards the 5xW reporter, whereas mutant Q174A showed the opposite, in which it promoted the 5xW reporter but no detectable activity on 6xCR4 reporter. Although E181 doesn’t interact directly with DNA, it interacts with Q157 and R150, both of which interact with DNA backbone, well explaining why E181A and Q157A showed the same defective function toward the two reporters (Fig. 3a). Similarly, although V269 doesn’t interact with DNA directly, it forms hydrogen bonds with N273; the latter interacts directly with base A in the PORE half sequence AAAT (Fig. 3a), explaining why V269A and N273A displayed the same pattern of defects in the reporter assays. Interestingly enough, Q174 also interacts directly with base A in the PORE sequence AGGCAAAT, underlying its defect in the 6xCR4 reporter, despite Q174A interacted very well with Sox2 as shown in the co-immunoprecipitation assay (Fig. 3e). Together these data strongly suggest that differential activation of Oct4 target genes displays differential requirements for its critical residues, defects of any kind of them would lead to the same phenotype during iPSC reprogramming.

### POU domain of Oct4 is the minimal requirement for generating iPS cells

**Figure 4:**
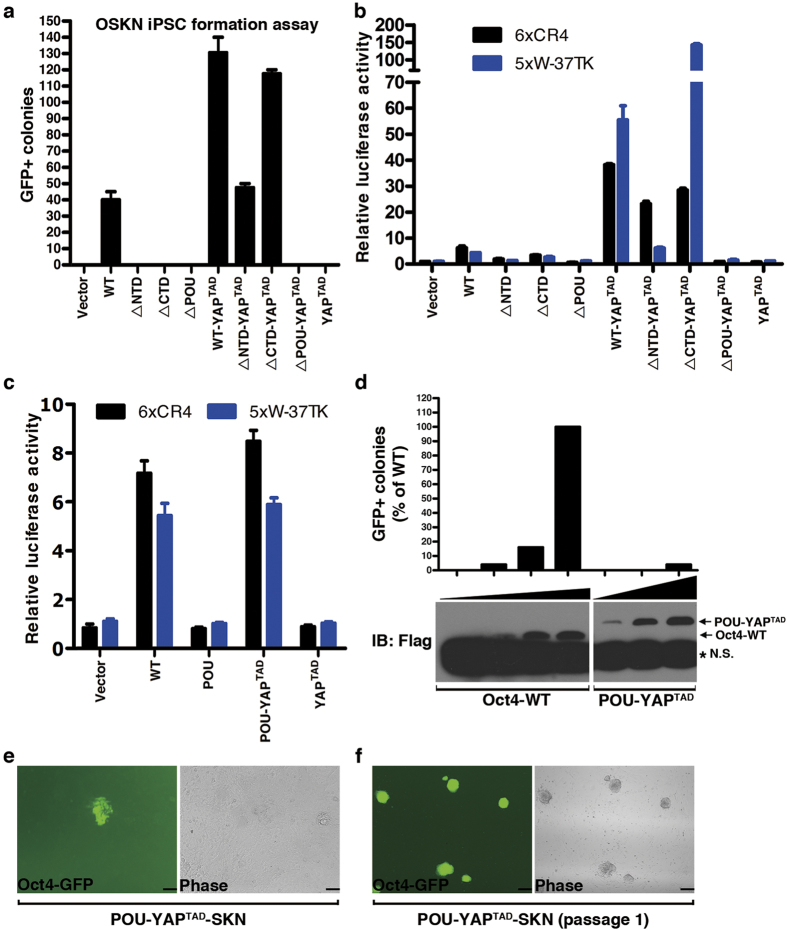
POU domain of Oct4 is the minimal requirement for generating iPS cells. (**a**) The number of GFP+ iPSCs colonies formed by mOct4 WT, the indicated deletion mutants, or the YAP^TAD^ fusion proteins was calculated. Data are representative of three independent experiments. Statistics analysis was performed using a t-test (mean and s.d. of duplicate assays). (**b**) The relative transcriptional activities of mOct4 WT, the indicated deletion mutants, or the YAP^TAD^ fusion proteins were measured using 6xCR4-Luc and 5xW-Luc reporters, respectively. Data are representative of at least three independent experiments. Statistics analysis was performed using a t-test (mean and s.d. of duplicate assays). (**c**) The relative transcriptional activities of mOct4 WT, POU, POU-YAP^TAD^ and YAP^TAD^ were measured using 6xCR4-Luc and 5xW-Luc reporters, respectively. Data are representative of at least three independent experiments. Statistics analysis was performed using a t-test (mean and s.d. of duplicate assays). (**d**) The number of GFP+ iPSC colonies formed by increasing doses of mOct4 WT and POU-YAP^TAD^ was calculated. The corresponding protein levels of Oct4 WT and POU-YAP^TAD^ were shown in the lower panel. *:non-specific band (N.S.). (**e**) iPSC colony formed by POU-YAP^TAD^ under GFP fluorescence and phase contrast. Scale bars, 100 μm. (**f**) Establishment of iPS cell lines generated by POU-YAP^TAD^. Scale bars, 250 μm.

Our mutagenesis screening revealed that none of the mutants located outside of the Oct4 POU domain showed significant defects in our reprograming assays. This promoted us to consider the possibility that for reprograming to succeed the minimal domain contributed from Oct4 could just be its POU domain. To test this, we first deleted the N-terminal domain (∆NTD), the C-terminal domain (∆CTD), or the POU domain (∆POU) (Supplementary Fig. S4a), and used them in the iPSC assay. None of these constructs showed detectable GFP+ colonies and transactivation activities in the reporter assays (Fig. 4a,b). Their lack of activities was not due to their defect in protein expression, which didn’t show significant difference from that of Oct4 WT (Supplementary Fig. S4b). Instead, these results suggested that both the NTD and the CTD of Oct4 are critical for Oct4 transcriptional activity. We then tested them after fusion to the transactivation domain (TAD) of YAP (YAP^TAD^) (∆NTD-YAP^TAD^, ∆CTD YAP^TAD^, ∆POU-YAP^TAD^). Our previous studies using YAP TAD domain fused to the reprogramming factors Oct4, Nanog and Sox2 have shown that YAP can dramatically enhance iPSC efficiency and more importantly, significantly accelerate the reprogramming kinetics^20^. As shown in Fig. 4a, fusion of YAP^TAD^ restored the reprogramming activities of ∆NTD and ∆CTD but not ∆POU constructs. These results are consistent and correlate very well with their relative transcriptional activities in the reporter assays (Fig. 4b). The finding that fusion of YAP TAD restored the transcriptional and reprogramming activities of Oct4 ∆NTD and ∆CTD implies that there could be no ESC-specific factor(s) to be recruited by the N- and C-terminal TADs to regulate induction of Oct4 target genes during somatic cell reprogramming. A previous work also showed that only the POU domain but neither the N- nor the C- terminal TADs is essential for the self-renewal of mouse ES cells^22^.

To further test the implication, we then focused on the POU domain alone. Not surprisingly, POU domain alone didn’t exhibit any transcriptional activities in the reporter assays (Fig. 4c). Interestingly, fusion of the POU domain to the YAP TAD domain (POU-YAP^TAD^) restored its transcriptional activity to the level comparable to that of Oct4 WT (Fig. 4c). Furthermore, POU-YAP^TAD^ formed GFP+ colonies in 16 days in the iPSC assay using the OSKN regime (Fig. 4d,e), which could be used to establish iPS cell lines (Fig. 4f). However, we should point out that the reprogramming efficiency of POU-YAP^TAD^ is lower than that of mOct4 WT. Transfection experiments in 293T cells showed POU-YAP^TAD^ expressed much lower amount of proteins compared to mOct4 WT (data not shown). Viral transduction into MEF cells also showed that, when using the same titers of recombinant viruses in the assay, the expression level of POU-YAP^TAD^ is much lower than that of mOct4 WT (Supplementary Fig. 4b). Together these data strongly show that for Oct4, although the POU domain is unique, the N- and C-terminal TADs are not unique and can be replaced with the TAD domain of YAP without much defect in terms of transcriptional activity. This may well explain why in our alanine screening assays we didn’t find any mutants outside of the POU domain that had strong defects in iPSC reprogramming.

### Critical residues confer Oct4 uniqueness in reprogramming

To further characterize specific residues that make Oct4 unique in reprogramming, we looked into the Oct4 POU domain sequences in more details. The POU domain of Oct4 family is extremely conserved and there are only seven highly variable residues that have been changed among the 12 Oct4 proteins from the 12 species (Supplementary Fig. 5a). Among which, S216, S237, T240, K244 are in our mutant collection. Not surprisingly, they didn’t show any defects in our reprogramming assay (Supplementary Fig. S1c), suggesting these residues are not under selection pressure during evolution. However, close examination of the 16 murine POU family members revealed that there are many residues that have been substituted compared to mOct4 across the whole POU domain (Supplementary Fig. 5b). The most enriched regions undergoing substitution are the linker regions connecting the POU_S_ and the POU_HD_ subdomains and the first 2 helices of the POU_HD_ subdomain. The α3-helix from both the POU_S_ and the POU_HD_ domains doesn’t change much, consistent with their critical functions in target gene DNA binding, in which both locate in the major grooves of the PORE DNA helix.

Among those residues undergoing heavy replacement, two of them, K170 and K215, caught our attention (Supplementary Fig. 5c). K215 is in the linker region of POU_S_ and POU_HD_ subdomains, which has been substituted by Ser, Thr, Leu, Pro or even Asp in other murine POU members (Supplementary Fig. S1e). The available crystal structure shows that K215 interacts with Q211; the latter also is in the linker region connecting POU_S_ and POU_HD_ subdomains. Q211 in turn interacts with Y155 (Fig. 2b). K215 also interacts with D159. So, through the unique K215, together with Q211, Y155 and D159 forming intra-molecular interactions, they cluster together to determine the uniqueness of Oct4 among the POU-containing protein family. Consistent with this, the three mutants, K215A, Y155A and D159A all lost their activity in the reprogramming assay (Fig. 1a). Although we have not tested Q211 mutant, Esch *et al.* showed that mOct4 Q211R mutant lost its capability in driving iPSC formation^5^.

**Figure 5:**
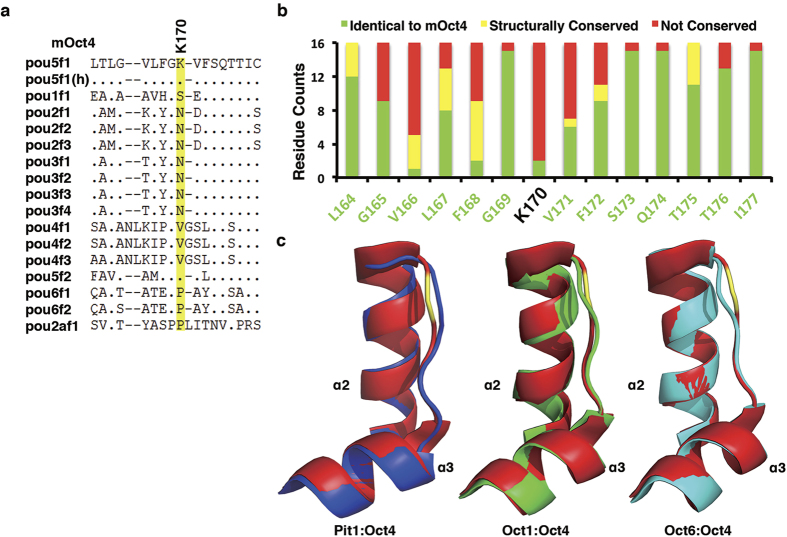
Specific residues make Oct4 unique in reprogramming. (**a**) Sequence alignment of various murine POU domains showing the unique K170 in Oct4, which is highlighted in yellow. (**b**) Residue substitution counts surrounding mOct4 K170 among the 16 murine POU domain-containing members. (**c**) Superimpositions of the POUs helices α2–α3 between Oct4 and Pit^23^, Oct1^24^ or Oct6^25^. The POUs helices α2–α3 of Oct4, Pit1, Oct1 and Oct6 are shown as cartoon in red, blue, green, and cyan, respectively. The unique K170 of Oct4 is highlighted in yellow.

As to the K170, it locates in the linker region of helices α2 and α3 of the POU_S_ subdomain. The 12 Oct4 proteins we sampled from 12 different species all are Lys at this position (Supplementary Fig. S1d), suggesting its importance for Oct4 function. Accordingly, changing K170 to Ala rendered Oct4 inactive in the iPSC reprogramming assay. However, this position has been substituted by Asn, Val, Pro and Ser, respectively, in other murine POU members, distinguishing Oct4 from other POU members (Fig. 5a,b). Superimposition of crystal structures of available POU members (Pit1^23^, Oct1^24^ and Oct6^25^) with Oct4 revealed that, although the helices α2 and α3 can be superimposed very well, the linkers in between containing K170 adopt different structures and display significant divergence (Fig. 5c). We would argue that K170 could be considered as another specific residue that makes Oct4 unique in the POU family. Functionally it is quite possible that this region interacts with some specific factors facilitating reprogramming.

To examine if the two sites are also unique in human Oct4 (hOct4), we made sequence alignment on all human POU domain-containing proteins (Supplementary Fig. S6a). As is the case in mOct4, hOct4 K177 (corresponding to mOct4 K170) and K222 (corresponding to mOct4 K215) are also highly specific to hOct4. We mutated them to Ala and tested their activities in the reprogramming assay. Not surprisingly, while hOct4 WT shows strong reprogramming activities, K177A almost lost its capacity in GFP+ colony formation and K222A gave rise to only about half of that of WT, which was exactly what has been seen with mOct4 K170A and K215A mutants (Supplementary Fig. 6b). Both mutants didn’t show detectable defects in protein expression when compared to hOct4 WT (Supplementary Fig. 6c). Taken together, we uncovered critical residues that are unique to Oct4, which confers Oct4 uniqueness in somatic cell reprogramming.

## Discussion

Oct4, discovered 25 years ago, has been well characterized from many different aspects to be the master transcription factor in regulating the self-renewal and pluripotency of ES cells in mouse, human and many other species. Oct4 is also the key transcription factor in the somatic cell reprogramming. Compared with its broadly characterized functions, such as the protein repertoires that Oct4 interacts with, the transcriptional networks that Oct4 regulates and its transcriptional regulations, the mechanisms through which that Oct4 itself, especially about its PTMs and uniqueness in the POU domain faimly, is regulated have largely been open for further exploration, although there have been studies about its phosphorylation, sumoylation, ubiquitination. In this study, we took an unbiased method and generated a library of Oct4 mutants that covered all the Ser, Thr, Tyr, Lys and Arg residues and characterized their activities in iPSC reprogramming assay, transactivation reporter assay, subcellular localization assay and protein stability assay. To our knowledge, this is the first of this kind that employed such strategy to discover critical residues for Oct4 functionality and specifying Oct4 uniqueness among the POU domain-containing protein family.

Our data uncovered many critical residues for Oct4 function, in which almost all of them are actually located within the POU domain of Oct4, presumably functioning as key residues in interaction with its cognate DNA sequences of its target gene promoters. This assumption is supported by the co-crystal structure studies of mOct4 and the PORE DNA sequence^5^. Our data also imply that whatever the PTMs on the residues outside of the POU domain are not essential for Oct4 function, although we could not exclude the possibility that PTMs could modulate Oct4 to fine-tune its functions for ES cell self-renewal and pluripotency, as have been reported for its phosphorylation and sumoylation. Wei *et al.* has shown that SUMO-1 modification of mOct4 at K118 (K123 in human Oct4) results in its increased stability, DNA binding, and transactivation, which provides an important mechanism to regulate Oct4 activity^11^. This residue locates at the end of the N-terminus of the transactivation domain of Oct4 and right before the POU domain. In our study, K118A mutant gave rise to a reprogramming efficiency of about 50% of that of Oct4 WT. This is actually a quite good consistency with the report by Wei *et al.*^11^, a verification of our assay. O-GlcNAc modification of mOct4 T228 regulates its transcriptional activity in ES cells and also plays important role during somatic cell reprogramming^10^. In human embryonic carcinoma cells (ECCs), T235 (corresponding to mOct4 T228) undergoes phosphorylation mediated by Akt, which results in enhanced protein stability, subcellular localization and transcriptional activities towards key stemness genes, thus promoting survival and tumorigenicity of ECCs^7^. In our work, mOct4 T228A showed reduced reprogramming activity but didn’t show detectable defects in protein stability and reporter activities. We should point out that one caveat of our Ala scanning assay is this kind of assay would miss those sites that might play negative regulatory roles if they undergo relevant modifications.

Numerous studies have shown Oct4 is unique among the POU family members and cannot be replaced by any other POU family members for successful iPS reprogramming^3^,^15^,^16^,^17^. However, among the critical residues we have characterized, most of them are extremely conserved in the Oct4 family during evolution and also among the POU family members from the same species such as mouse and human, making it difficult to understand the basis of Oct4 uniqueness in reprogramming. Esch *et al.* reported that the first half of the linker region of murine Oct4 connecting the POU_S_ and POU_HD_ domains forms a unique interface different from other four POU members with available crystal structures, which can partly explain Oct4 uniqueness^5^. Here we also characterized two of the critical K residues among all the S/T/Y/K/R residues, which are unique to Oct4 among all the mouse and human POU family members, to explain, at least in part, the source of the Oct4 uniqueness, adding more insights into our understanding about Oct4 function in reprogramming. It is highly possible some of the residues other than S/T/Y/K/R that we have not studied could also contribute to Oct4 uniqueness in iPS reprogramming.

Although reprogramming to pluripotency by combination of Oct4 with other factors has been successful from different types of somatic cells, the underlying molecular mechanisms remain poorly understood. Overcoming of epigenetic barriers, induction of ES cell-specific pluripotency genes, repression of somatic cell differentiation genes, and induction of mesenchymal-to-epithelial transition (MET) are events that have been proposed for a successful somatic cell reprogramming^3^. During the process, Oct4 protein level has been proposed as an important parameter, especially at the early stages of the reprogramming process^26^. In support of this notion, our mutant library has 4 mutants that are not as stable as Oct4 WT showing much reduced expression levels and so these mutants lost activities in our reprogramming assays.

Our fusion studies with the YAP TAD domain are kind of surprising at the first glance. The Yes-associated protein (YAP) has been established as an important transcriptional coactivator with a potent transactivation domain (TAD) in its C-terminus. Studies have shown that YAP also plays a critical role in maintenance of stem cell pluripotency^27^. Ectopic expression of YAP promotes cell growth and induces tumor formation^28^. Our recent finding that fusion of YAP TAD domain with the generic iPS reprogramming factors Oct4, SOX2 and Nanog could induce highly efficient somatic cell reprogramming towards iPS cells by dramatic enhancement of TET1 induction at the early reprograming stage^20^. Here in this report, we showed that fusion of the YAP TAD domain with the Oct4 POU domain could replace the full-length Oct4 in the reprogramming process. This result implies the POU domain is the minimal requirement for Oct4 function in iPSC, which might bind to ES cell specific regulatory factors^29^. This is consistent with the fact that across Oct4, only the POU domain is evolutionary conserved whereas the N- and C-termini are highly variable among the POU family proteins. What the true functions are for the N- and C-termini or the YAP TAD domain need further investigation. They either play positive roles by recruiting other general transcriptional factors, co-factors or even epigenetic enzymes, or play negative roles by repressing negative factors such as p53. It has been shown by several studies that p53 is a key negative regulator in iPS reprogramming or generation of ES-like cells from neonatal and adult testis^30^. However, these fusion studies are in good support to the “stochastic” model of somatic cell reprogramming, during which reprogramming factors such as Oct4 bind the genome in a promiscuous way acting as “pioneer” factors to open up the repressed chromatin and recruit other transcriptional activators, chromatin remodelers and the transcriptional machinery^31^,^32^. In other words, at the very early stage of reprogramming, the specificity of the TAD domain of Oct4 is not critical and so any potent TADs such as YAP could be used to support reprogramming.

Our current assay has not characterized the post-reprogramming events after endogenous Oct4 is induced, which can then replace recombinant Oct4 while its expression is being silenced along iPS progression. During the late stage of the reprogramming, after the induction of full-length Oct4 from endogenous Oct4 loci, the exogenous expression of Oct4 may no longer be needed for the reprogramming process, which should be silenced along the reprogramming progression. So for the target gene expression, does it mean the cofactors that could be defined by the N- and/or C-termini of Oct4 to determine the specificity and/or efficiency is not that important for ES cell self-renewal and maintenance of pluripotency. It would be very interesting to see if the Oct4^POU^-YAP^TAD^ can replace and function like endogenous Oct4 for the self-renewal and pluripotency of EC cells and iPS cells.

In summary, in this study, we took an unbiased method and generated a library of Oct4 mutants that covered all the Ser, Thr, Tyr, Lys and Arg residues and characterized their activities in iPSC reprogramming assay. We uncovered many critical residues for Oct4 function, many of which are actually located within the POU domain of Oct4 functioning as key residues in interaction with its cognate DNA. Although the N- and C-terminal TADs are critical for Oct4 function, they are not unique to Oct4 and can be functionally substituted by TADs from other proteins such as YAP. But the POU domain contains unique residues that confer Oct4 uniqueness, making it special in the POU family. Motifs containing these unique residues would recruit specific factors important for somatic cell reprogramming. Identification of such specific factors would provide more insightful understanding of Oct4 unique function during reprogramming and even its uniqueness in the self-renewal and pluripotency of ES cells.

## Materials and Methods

### Cell culture

HEK293 and HeLa cells were cultured in high-glucose Dulbecco’s modified Eagle’s medium (DMED; Gibco) supplemented with 10% FBS (Hyclone) and antibiotics (100 U/ml penicillin and 100 μg/ml streptomycin; Gibco). For MEFs, the cell culture medium consists of 10% FBS (Hyclone), 1 mM sodium pyruvate (Gibco), 0.055 mM β-mercaptoethanol (Gibco), 2 mM L-GlutaMax (Gibco), 0.1 mM NEAA (Gibco), and antibiotics (100 U/ml penicillin and 100 μg/ml streptomycin; Gibco). For iPSC generation cell culture media, 1000 U/ml LIF (Millipore) were added based on MEF media. iPSC stable clones were maintained on mitomycin C-treated MEF feeder cells in knockout DMEM medium (Gibco) supplemented with 15% KSR, 1 mM sodium pyruvate (Gibco), 0.055 mM β-mercaptoethanol (Gibco), 2 mM L-GlutaMax (Gibco), 0.1 mM NEAA (Gibco), 100 U/ml penicillin (Gibco), 100 μg/ml streptomycin (Gibco) and 1000 U/ml LIF (Millipore). CGR8 ES cells were cultured on gelatin-coated dishes, in GMEM medium supplemented with 10% FBS (Millipore), 1 mM sodium pyruvate (Gibco), 0.055 mM β-mercaptoethanol (Gibco), 2 mM L-GlutaMax (Gibco), 0.1 mM NEAA (Gibco), 100 U/ml penicillin (Gibco), 100 μg/ml streptomycin (Gibco) and 1000 U/ml LIF (Millipore). P19 EC cells were maintained on gelatin-coated dishes, in α-MEM medium supplemented with 10% FBS (Hyclone) and 100 U/ml penicillin (Gibco), 100 μg/ml streptomycin (Gibco).

### Antibodies

The antibodies used in this study included mouse anti-Flag (Sigma, 1:3000), rabbit anti-Flag (Sigma, 1:2000), rabbit anti-Myc (MBL, 1:3000), mouse anti-GFP (Abmart, 1:5000), mouse anti-GAPDH (Sungene Biotech, 1:5000), mouse anti-α-Tubulin (Sungene Biotech, 1:5000), rabbit-anti-Oct4 (Santa Cruz, 1:2000), rabbit anti-HA (MBL, 1:3000), HRP-conjugated goat anti-mouse (Sigma, 1:3000), HRP-conjugated goat anti-rabbit (Sigma, 1:3000), goat anti-mouse Alexa555 (Molecular Probes, 1:2000) and goat anti-mouse Alexa488 (Molecular Probes, 1:2000).

### Plasmid construction

#### Generation of the murine Oct4 mutant library

pMXs-*Oct4* (O), pMXs-*Sox2* (S), pMXs-*Nanog* (N), pMXs*-c-Myc* (M) and pMXs-*KLF4* (K) retroviral vectors were purchased from Addgene. CHR-ccdB fragment was amplified from pLX304, and cloned into pMXs-retroviral vector to create pMXs-CHR-ccdB destination vector. To generate pDONR223-*Oct4*-Flag entry clone, the cDNA sequence of murine Oct4 was amplified from pMXs-*Oct4*, and was cloned into pDONR223 donor vector. Mutants were generated by PCR-based site-directed mutagenesis on pDONR223-*Oct4* entry clone. All Oct4 entry clones were verified by sequencing and transferred into pMXs-CHR-ccdB destination vector by LR recombination reaction to generate Oct4 mutant library.

#### Other vectors construction

pGL4.2-6xCR4 was previously described by Zhu *et al.*^20^. −37TK promoter was amplified from pRL-TK, and cloned into pGL4.2-Basic vector to create pGL4.2-37TK. W oligonucleotide sequences were previously described by Hans R.Schöler *et al.*^33^. W oligonucleotides containing CCC in the 5′ and GGG in the 3′ were annealed, then subcloned into pGL4.2-37TK to create pGL4.2-5xW-37TK. Sox2 fragment was amplified from pMXs-*Sox2* and cloned into pMXs-retroviral vector to create pMXs-*Sox2*-Myc. EGFP fragment was amplified from pCS-EGFP and cloned into pMXs-retroviral vector to create pMXs-EGFP-Flag. ΔNTD, ΔCTD, ΔPOU and POU fragments were amplified from pMXs-*Oct4*, and subsequently cloned into pMXs-retroviral vectors to create Oct4 deletions vectors (O^deletion^). ΔNTD, ΔCTD, ΔPOU and POU fragments amplified from pMXs-*Oct4* were fused with TAD domain of mouse YAP in the C-terminal to generate ΔNTD-YAP^TAD^, ΔCTD-YAP^TAD^, ΔPOU-YAP^TAD^ and POU-YAP^TAD^. These fused fragments were cloned into pMXs-retroviral vectors and named O^deletion^y. pMXs-*Oct4-*WT-YAP^TAD^ (Oy) was previously described by Zhu *et al.*^20^. YAP^TAD^ was amplified from Oy and cloned into pMXs-retroviral vector. Human Oct4-WT-Flag was amplified from pMXs-*hOct4* (purchased from Addgene), and cloned into pMXs-retroviral vector to generate pMXs-*hOct4*-WT-Flag. pMXs*-hOct4*-K177A and pMXs*-hOct4*-K222A mutants were generated by PCR-based site-directed mutagenesis on pMXs-*hOct4*-WT-Flag.

### Retrovirus production and mouse iPSCs induction

For retrovirus production, pMXs retroviral vector together with packaging plasmid Ecopac (1:1) were cotransfected into HEK293 cells. At 8–12 h post-transfection, the medium was replaced. At approximately 56 h post-transfection, the virus supernatant was collected and filtered through a 0.45 μm polyvinyl difluoride (PVDF) filter (Millipore). OG2-MEFs were seeded at 2.5 × 10^4^ cells per well in a 12-well plate at 1 day before infection. The several kinds of viruses cocktail (OSK, OSKM, OSKN, OySKM, OySKN, O^deletion^SKM, O^deletion^SKN, O^deletion^ySKM and O^deletion^ySKN) were added at a ratio of 1:1:1 or 1:1:1:1 supplemented with 8 μg/ml polybrene. At 24 h post-infection, the virus supernatant was replaced with MEF medium. At day 4 post-infection, cell culture medium was replaced by iPSC medium until iPSCs colonies generation. For OSKM or OSKN-iPSCs generation, at approximately days 12–16, Oct4-GFP expression was observed and Oct4-GFP-positive iPSCs colonies appeared. Oct4-GFP-positive iPSCs colonies were then counted at days 16–20. For OySKM or OySKN iPSCs generation, Oct4-GFP expression was observed at days 6–10 and iPSCs colonies number was counted at days 10–14. iPSCs colonies usually were picked after GFP-positive clones were counted.

### AP staining

For AP staining, cells were stained using an Alkaline Phosphatase Detection kit (SCR004; Millipore) according to the manufacturer’s protocols. OSKM-iPSCs were stained for AP between days 16–20 post-infection.

### Crystal structure analysis and modeling

Oct4^POU^:PORE complex crystal structure (3L1P) was analyzed using Pymol software. In general, we focused on residues which showed strong defects in our reprogramming experiments. Firstly, in 3L1P crystal structure, we found that side chains of K170, K188, K192, K215, K224, R225, K226, K277 and K279 are missing. Based on highly conserved POU domain between POU family members and available 1HF0 (Oct1^POU^:PORE) crystal structure, we aligned 3L1P and 1HF0 in Pymol and then modeled new side chains, in the model of Oct4^POU^:PORE complex, of K188, K192, K226, K277 and K279 according to the superposition. Secondly, we set PORE-DNA as a unit to find out any amino acid which interacts with DNA through hydrogen bond, salt bridge or non polar contacts in the model of Oct4^POU^:PORE complex. Thirdly, based on our reprogramming screening result, we set a functional residue as a target amino acid each time to find out other amino acids which interact with the target amino acid through hydrogen bond, salt bridge or non polar contacts in 3L1P crystal structure.

### Reporter assay

For pGL4.2-6xCR4 reporter assay, the 6xCR4 luciferase reporter plasmid and pMXs*-Sox*2-WT were cotransfected into HEK293 cells together with a mock vector, a pMXs-*Oct4* WT or its mutants. As for pGL4.2-5xW-37TK reporter assay, the 5xW luciferase reporter plasmid was transfected into HEK293 cells together with a mock vector, a pMXs-*Oct4* WT or its mutants. In addition, a pRL-CMV renilla reporter plasmid was included in all transfection for normalization. At 36 hours post-transfection, cells were lysed for the measurement of luciferase and renilla activity. The relative fold of Oct4-induced luciferase activity was normalized with renilla activity.

### Coimmunoprecipitation

For coimmunoprecipitation assays, HEK293 cells were transfected by using pMXs-*Sox2*-Myc together with pMXs-EGFP-Flag, pMXs-*Oct4*-WT-Flag or pMXs-*Oct4*-Q174A-Flag. At 36 hours after transfection, cells were lysed in lysis buffer (50 mM Tris-HCl pH 7.5, 150 mM NaCl, 1 mM EDTA, 10% glycerol, 0.5% Triton X-100) supplemented with a protease-inhibitor cocktail (Invitrogen) on ice for 30 min, sonicated briefly, then clarified supernatants were incubated with anti-Flag (Sigma) beads. After pull-down for 4–6 h, the beads were extensively washed with washing buffer (50 mM Tris-HCl pH 7.5, 300 mM NaCl, 1 mM EDTA, 0.5% Triton X-100, 10% glycerol) three times for a total of 30 min. Samples were then subjected to Western blot analysis.

### Protein stability assay in MEF cells

MEFs were infected by pre-prepared virus cocktail (O^wt^ or O^mu^:S:K:M = 1:1:1:1) supplemented with 8 μg/ml polybrene for 24 h. Cells were collected and directly lysed in 2x Loading Buffer 5 days post infection. WB analysis was performed to detect protein stability of Oct4 WT or its mutants.

### CHX-based assay and WB band intensity analysis

HEK293 cells were transfected by using pMXs*-Oct4* WT or mutants together with pCS-EGFP. After approximately third-six hours, cells were treated with CHX (20 μg/ml final concentration) for 0, 2, 4, or 6 h. Then Cells were directly lysed in 2x Loading Buffer and subjected to Western Blot analysis. WB band intensity was analyzed by Quantity One software. Oct4 band intensity was normalized based on the GFP band intensity.

### MG132 and chloroquine-based assay and WB band intensity analysis

pMXs-*Oct4* WT or mutants along with pCS*-*EGFP were cotransfected into HEK293 cells. After approximately third-six hours, cells were treated with DMSO, MG132 (50 μM final concentration), chloroquine (50 μg/ml final concentration), or MG132 plus chloroquine for another six hours. Then cells were lysed in 2x Loading Buffer and subjected to Western Blot analysis. WB band intensity was analyzed by Quantity One software. Oct4 band intensity was normalized based on the GFP band intensity.

### *In vivo* ubiquitination assays

pMXs*-Oct4* WT or mutants together with pEF-HA*-Ub* were cotransfected into HEK293 cells. At 36 h post-transfection, cells were treated with MG132 (50 μM final concentration) for another 6 h. Cells were lysed in lysis buffer (50 mM Tris-HCl pH 7.5, 150 mM NaCl, 1 mM EDTA, 0.5% NP-40, 10% glycerol, 1% SDS) supplemented with a protease inhibitor cocktail (Invitrogen). Samples were sonicated twice at 30% AMP for 5 seconds. Supernatant was collected by centrifugation at 13,000 rpm for 10 min at 4 °C. Before immunoprecipitation with anti-Flag beads (sigma), SDS concentration in the lysate was adjusted to 0.2%. After pull-down for 4–6 h, the beads were extensively washed with washing buffer (50 mM Tris-HCl pH 7.5, 500 mM NaCl, 0.5% NP-40, 10% glycerol, 1mM EDTA) three times for a total of 30 min. Samples were then subjected to Western blot analysis.

### Western blot analysis

WB samples were prepared according to different kinds of experiments. Samples were run on 12% SDS-PAGE gels. Following transferring, NC membranes were blocked for 30 min with 5% nonfat milk in PBS containing 0.5% Tween-20 (PBST) and incubated for 2 h at RT or overnight at 4 °C with primary antibody. Then the blots were washed three times for 10 min each with PBST, and incubated with secondary antibodies for 1 h at RT. The blots were washed again three times for a total of 30 min with PBST, and lastly exposed with ECL reagent (Pierce).

### Immunofluorescence

HeLa cells were cultured on glass coverslips and transfected with pMXs-*Oct4*-WT or mutants. At 36 h post-transfection, cells were fixed with 4% paraformaldehyde for 15 min, then fixed cells were permeabilized and blocked with 5% bovine serum albumin and 0.2% Triton-X100 for 30 min at RT. Cells were then incubated with primary antibody for 2 h. Fixed cells were washed three times for a total of 30 min with PBST and then incubated with secondary antibodies for 1 h. Nuclei were counterstained with Hoechst (Sigma: 1:2000). Lastly, fixed cells were washed again and imaging of the cells was performed using Zeiss LSM 719 META laser scanning confocal system.

## Additional Information

**How to cite this article**: Jin, W. *et al.* Critical POU domain residues confer Oct4 uniqueness in somatic cell reprogramming. *Sci. Rep.* **6**, 20818; doi: 10.1038/srep20818 (2016).

## Supplementary Material

Supplementary Information
