# Supplementary material for: Critical POU domain residues confer Oct4 uniqueness in somatic cell reprogramming
Source: Sci Rep. 2016 Feb 15;6:20818. doi: 10.1038/srep20818 (PMC4753506; doi:10.1038/srep20818)
Supplement: Supplementary Information [file srep20818-s1.pdf]

Supplementary information

Critical POU domain residues confer Oct4 uniqueness in somatic cell reprogramming

Wensong Jin[1, 2, 4], Lei Wang[3, 4], Fei Zhu[1], Weiqi Tan[2], Wei Lin[1], Dahua Chen[2, *], Qinmiao Sun[1, *] and Zongping Xia[3, *]

[1]State Key Laboratory of Membrane Biology, Institute of Zoology, Chinese Academy of Sciences, Chaoyang, Beijing 100101, P. R. China.

[2]State Key Laboratory of Stem Cell and Reproductive Biology, Institute of Zoology, Chinese Academy of Sciences, Chaoyang, Beijing 100101, P. R. China.

[3]Life Sciences Institute and Innovation Center for Cell Signaling Network, Zhejiang University, Hangzhou, Zhejiang 310058, P. R. China.

[4]These authors contributed equally to this work.

[*]**Authors to whom proofs should be addressed:**

Dahua Chen, PhD

Tel: 86-10-64807325

E-mail: chendh@ioz.ac.cn

Qinmiao Sun, PhD

Tel: 86-10-64807391

E-mail: qinmiaosun@ioz.ac.cn

Zongping Xia, PhD

Tel: 86-571-88981625

E-mail: xia2010@zju.edu.cn

Supplementary figures

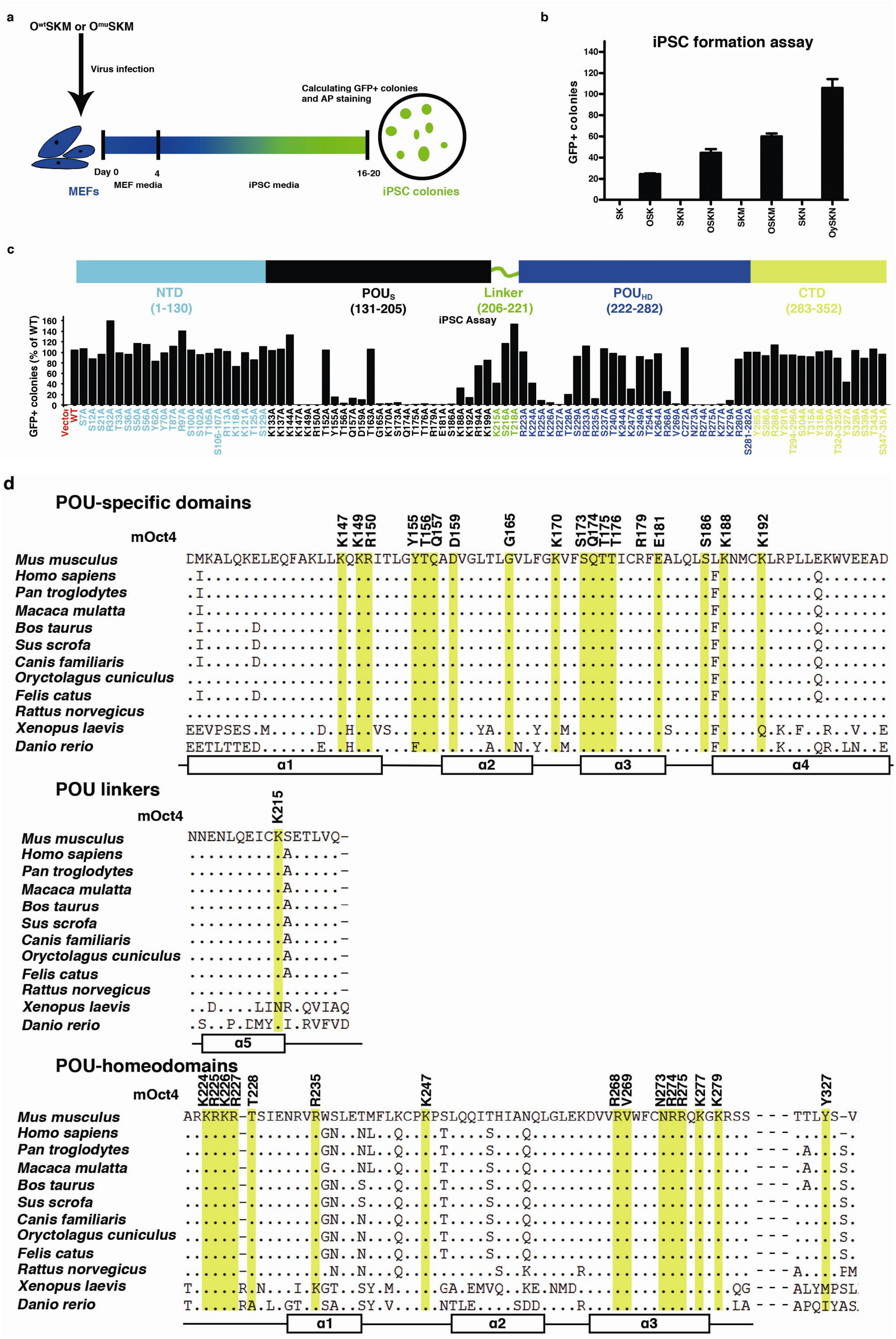

e

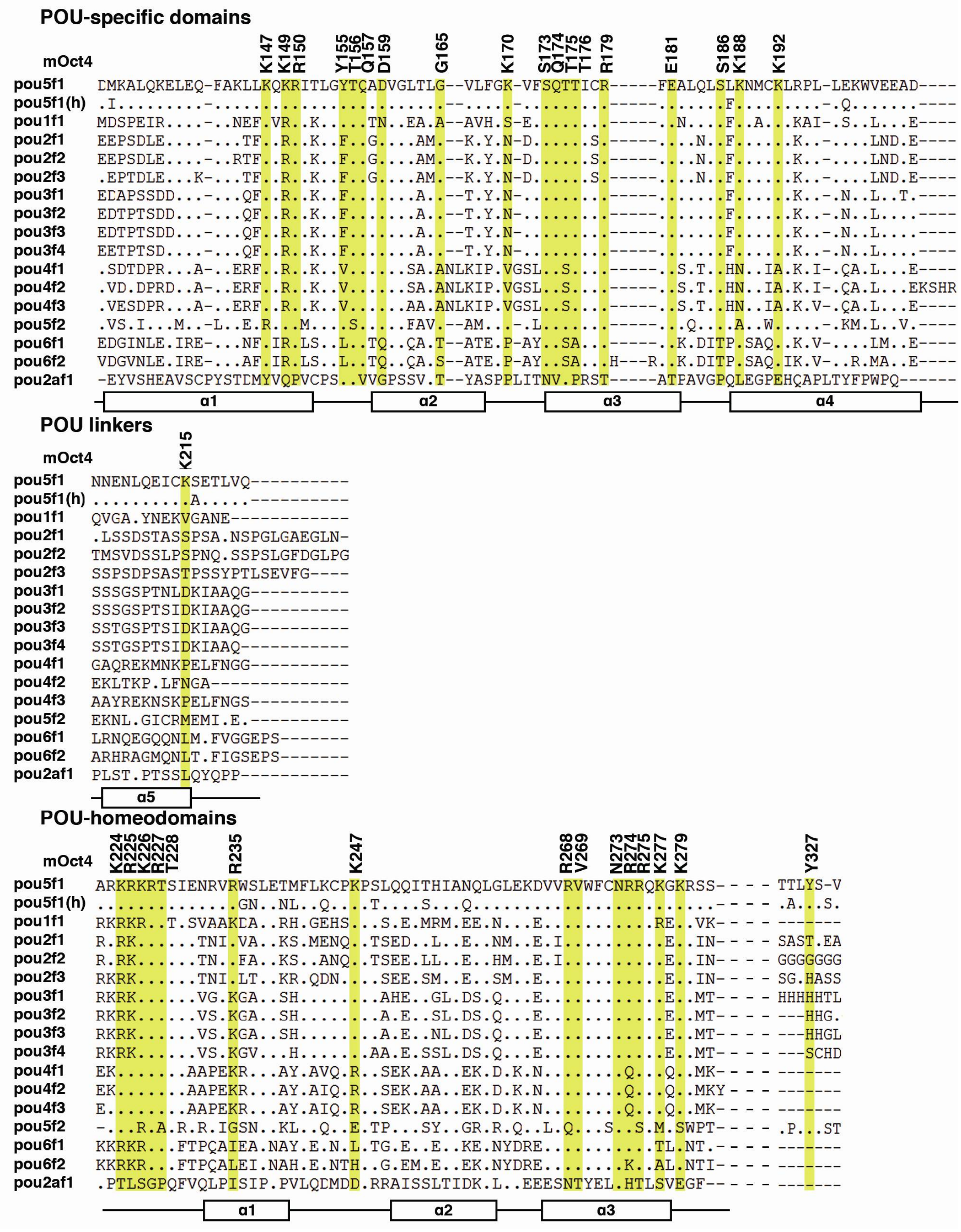
POU-specific domains
mOct4
K147 K149 R150 Y155 T156 Q157 D159 G165 K170 S173 Q174 T175 T176 R179 E181 S186 K188 K192
α1
α2
α3
α4
POU linkers
mOct4
K215
α5
POU-homeodomains
mOct4
K224 R225 K226 R227 T228 R235 K247 R268 V269 N273 R274 R275 K277 K279 Y327
α1
α2
α3

f

H-bond / AA / DNA	K147	K192	K247	K277	K279	R150	R179	R227	R268	R275
backbone	√	√	√	√	√	√			√	√
base							√	√		

g

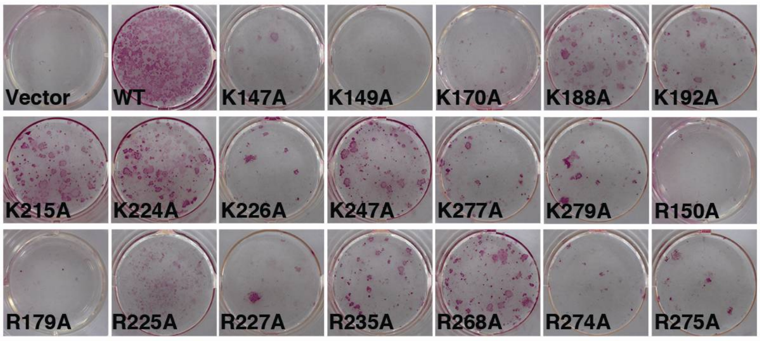

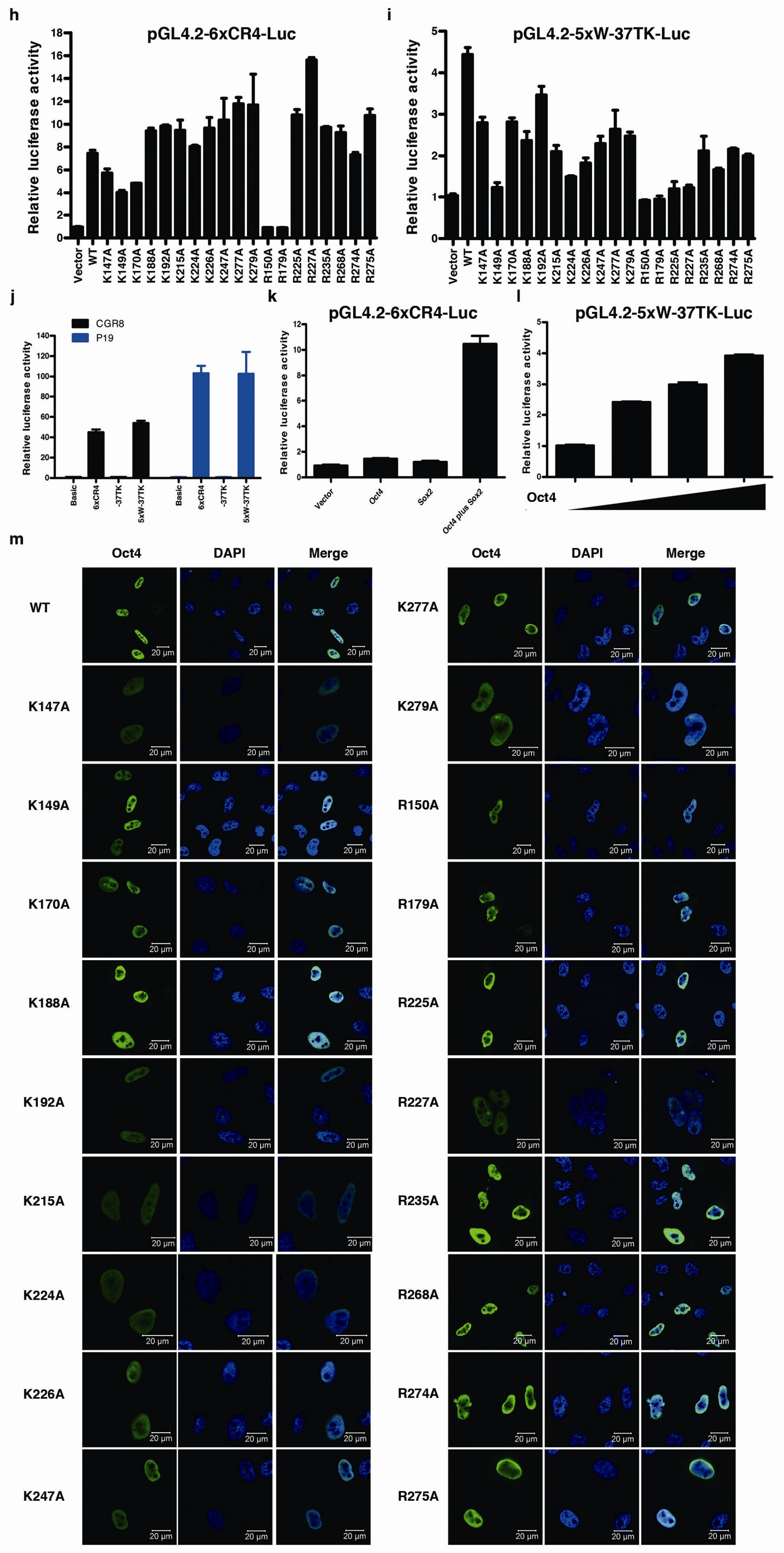
h
pGL4.2-6xCR4-Luc
Relative luciferase activity
Vector WT K147A K149A K170A K188A K192A K215A K224A K226A K247A K277A K279A R150A R179A R225A R227A R235A R268A R274A R275A
i
pGL4.2-5xW-37TK-Luc
Relative luciferase activity
j
CGR8
P19
Relative luciferase activity
Basic 6xCR4 -37TK 5xW-37TK Basic 6xCR4 -37TK 5xW-37TK
k
pGL4.2-6xCR4-Luc
Relative luciferase activity
Vector Oct4 Sox2 Oct4 plus Sox2
l
pGL4.2-5xW-37TK-Luc
Relative luciferase activity
Oct4
m
Oct4 DAPI Merge
WT K147A K149A K170A K188A K192A K215A K224A K226A K247A
K277A K279A R150A R179A R225A R227A R235A R268A R274A R275A
20 μm

Supplementary Figure S1 Identification of critical Oct4 residues for somatic cell reprogramming by alanine scanning.

(**a**) Schematic of experimental designs for library construction of mOct4 mutants and identification of the functional residues required for reprogramming activity.

(**b**) GFP+ iPSC formation by different combination of reprogramming factors. Data are representative of two independent experiments. Statistics analysis was performed using a t-test (mean and s.d. of duplicate assays).

(**c**) Percentage of GFP+ iPSC formation by the 90 mOct4 alanine mutants in the library. Data are representative of at least two independent reprogramming experiments for each mutant.

(**d**) Sequence alignment of 12 different Oct4 POU proteins. Residues that are defective in reprogramming after mutating to alanine are highlighted in yellow.

(**e**) Sequence alignment of 16 different murine POU proteins. Residues that are defective in reprogramming after mutating to alanine are highlighted in yellow.

(**f**) Table to show the interaction between K/R residues and DNA in Figure 1b.

(**g**) AP staining of OSKM-based reprogramming defective Oct4 K-to-A and R-to-A mutants.

(**h** and **i**) The relative transcriptional activities of reprogramming defective K-to-A and R-to-A mutants were measured using 6xCR4-Luc and 5xW-Luc reporters, respectively. Data are representative of at least three independent experiments. Statistics analysis was performed using a t-test (mean and s.d. of duplicate assays).

(**j**) Test of the 6xCR4-Luc or 5xW-Luc reporters in CGR8 ES cells and P19 EC cells. The Basic or -37TK luciferase enhancer-less vectors were served as negative controls.

(**k**) Test of the 6xCR4-Luc in HEK293 cells. 6xCR4-Luc was transfected into HEK293 cells together with a mock vector, Oct4, Sox2, or Oct4 plus Sox2.

(**l**) Test of 5xW-Luc in HEK293 cells. 5xW-Luc was transfected into HEK293 cells together with a mock vector or increasing amounts of Oct4. A pRL-CMV renilla reporter was included in all transfection for normalization. Data (**j**, **k** and **l**) are representative of three independent experiments. Statistics analysis was performed using a t-test (mean and s.d. of duplicate assays).

(**m**) Nuclear localization of reprogramming defective mOct4 K-to-A and R-to-A mutants. K-to-A and R-to-A mutants were transfected into HeLa cells grown on coverslips. Immunostaining of Oct4 was performed 36 h after transfection with DAPI counterstaining.

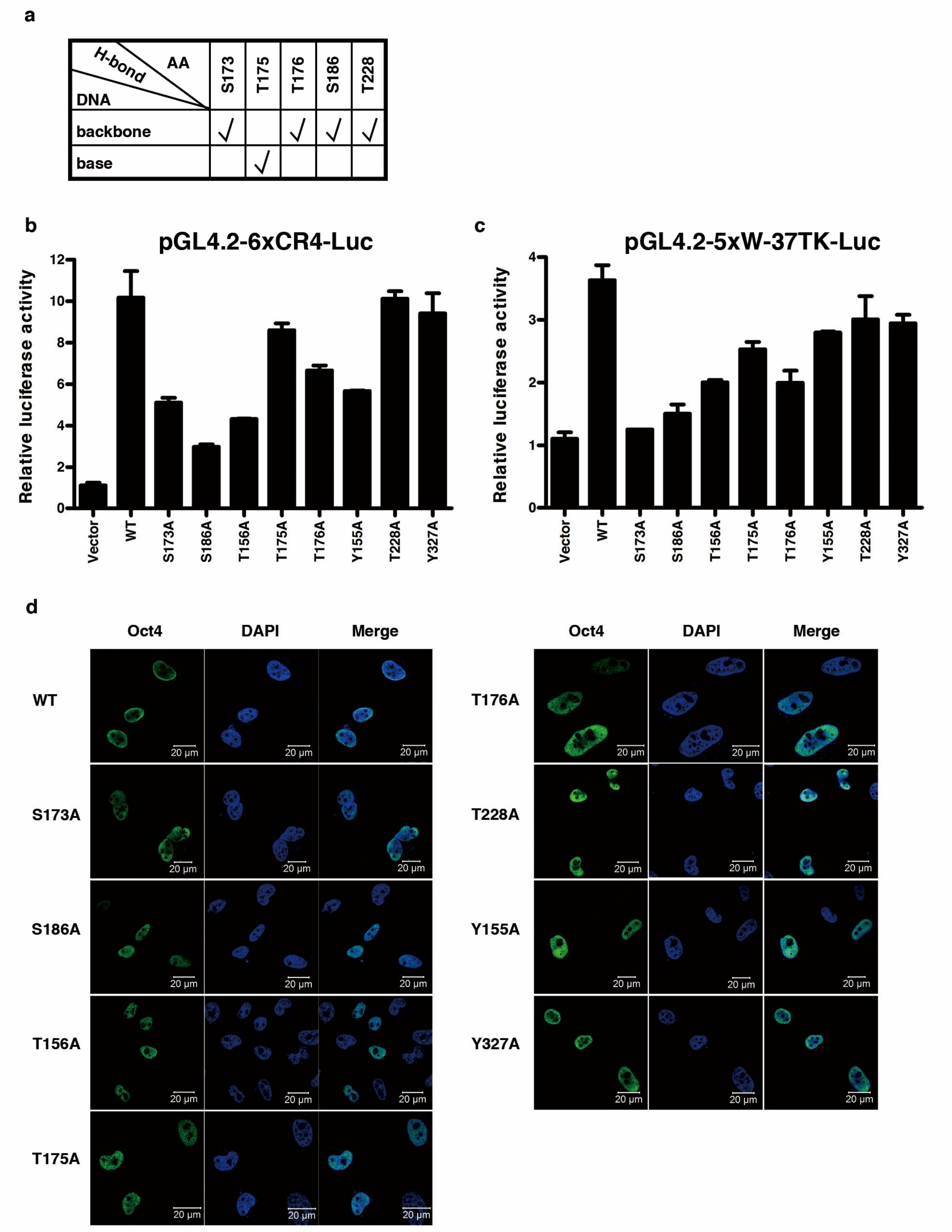

Supplementary Figure S2 Characterization of reprogramming defective mOct4 S-to-A, T-to-A and Y-to-A mutants.

(**a**) Table to show the interaction between S/T/Y residues and DNA in Figure 2b.

(**b** and **c**) The relative transcriptional activities of reprogramming defective S-to-A, T-to-A and Y-to-A mutants were measured using 6xCR4-Luc and 5xW-Luc reporters,

respectively. Data are representative of at least three independent experiments. Statistics analysis was performed using a t-test (mean and s.d. of duplicate assays).

(**d**) Nuclear localization of reprogramming defective mOct4 S-to-A, T-to-A and Y-to-A mutants. The mutants were transfected into HeLa cells grown on coverslips. Immunostaining of Oct4 was performed 36 h after transfection with DAPI counterstaining.

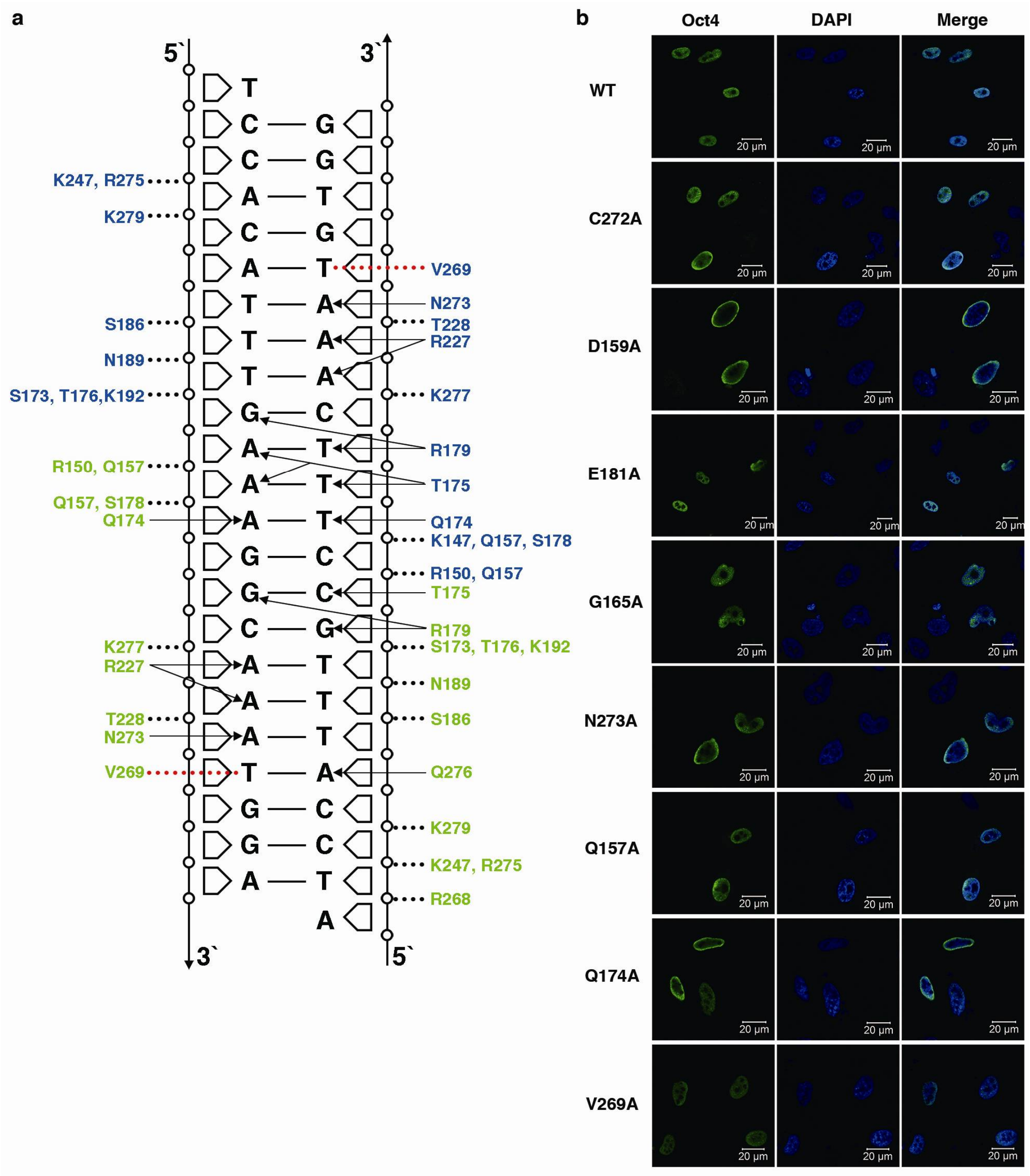

Supplementary Figure S3 Characterization of mOct4 critical DNA binding residues. (**a**) A sketch showing interactions or non-polar contacts between mOct4 residues and DNA in model of Oct4^{POU}:PORE complex. In the figure, interacting residues within the chain A (3L1P) are in blue, while interacting residues within the chain B are in green. Hydrogen bonds are shown by black dot lines for residues interacting with DNA phosphate and by solid arrows for those with DNA base. Non-polar contacts are shown by red dot lines.

(**b**) Nuclear localization of reprogramming defective mOct4 mutants. The mutants were transfected into HeLa cells grown on coverslips. Immunostaining of Oct4 was performed 36 h after transfection with DAPI counterstaining.

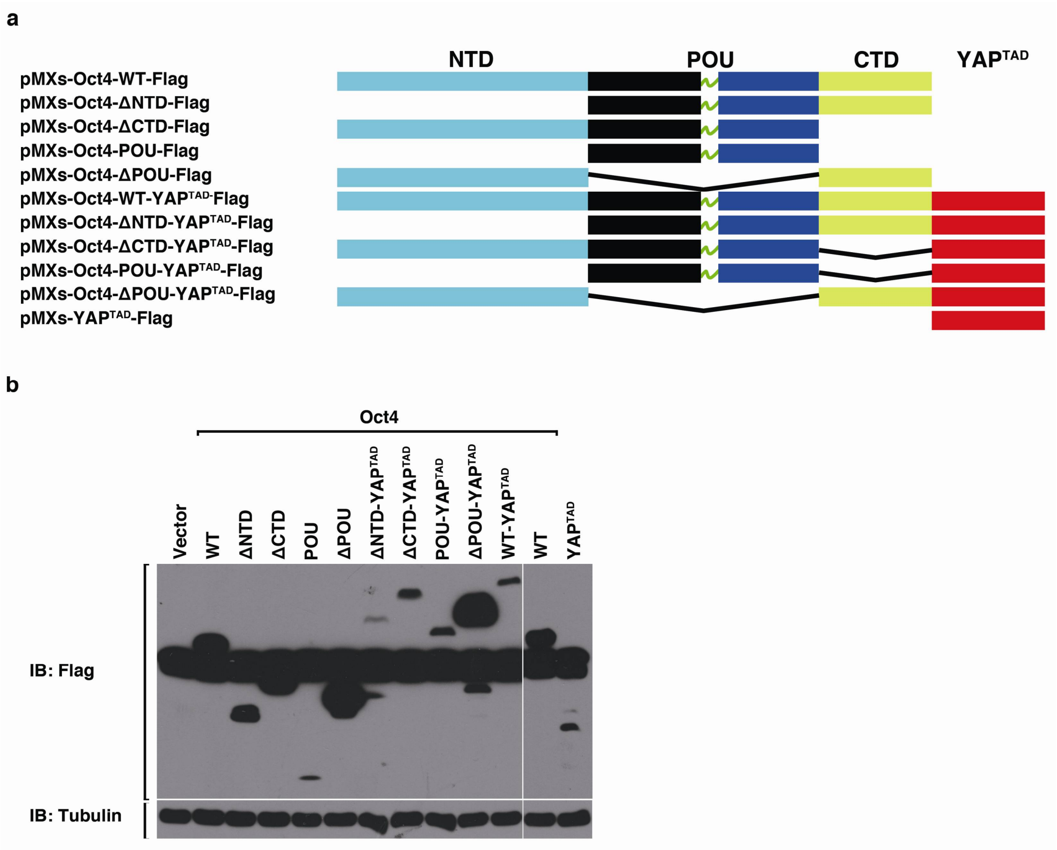

Supplementary Figure S4 POU domain of Oct4 is the minimal requirement for generating iPS cells.

(**a**) Diagrams of Oct4 WT, WT-YAP^{TAD} and their deletion constructs.

(**b**) Protein stability of Oct4 WT, WT-YAP^{TAD} and their deletion constructs in MEFs.

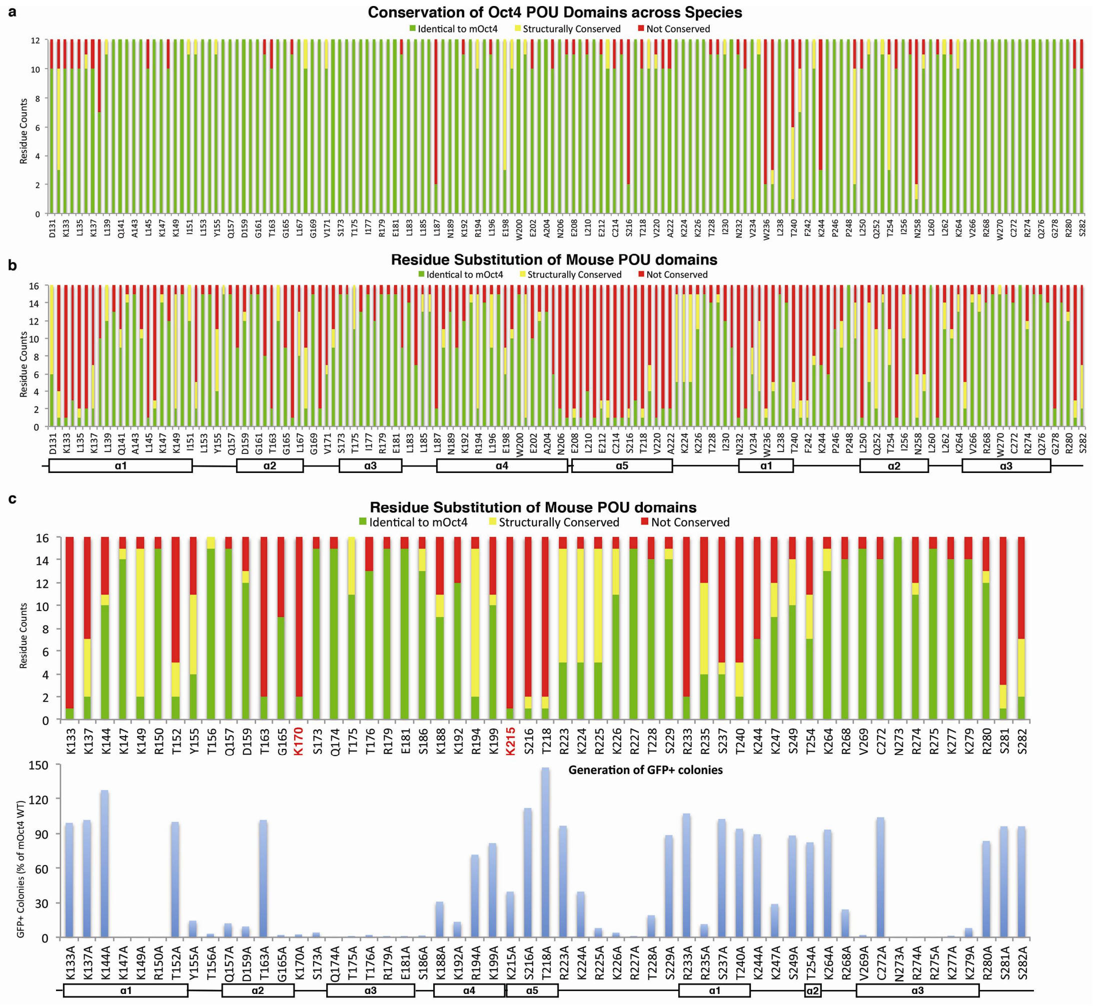

Supplementary Figure S5 Specific residues make Oct4 unique in reprogramming.

(**a**) Conservation of Oct4 POU domains across 12 species. The numbering is in accord with mOct4.

(**b**) Residue substitution among the 16 murine POU domain-containing members. The numbering is in accord with mOct4.

(**c**) Substitution of residues tested in our alanine mutant library collection among the 16 murine POU domain-containing members. For easier viewing, the effects of the Oct4 mutants in iPSC assay are shown in the bottom panel. The numbering is in accord with mOct4.

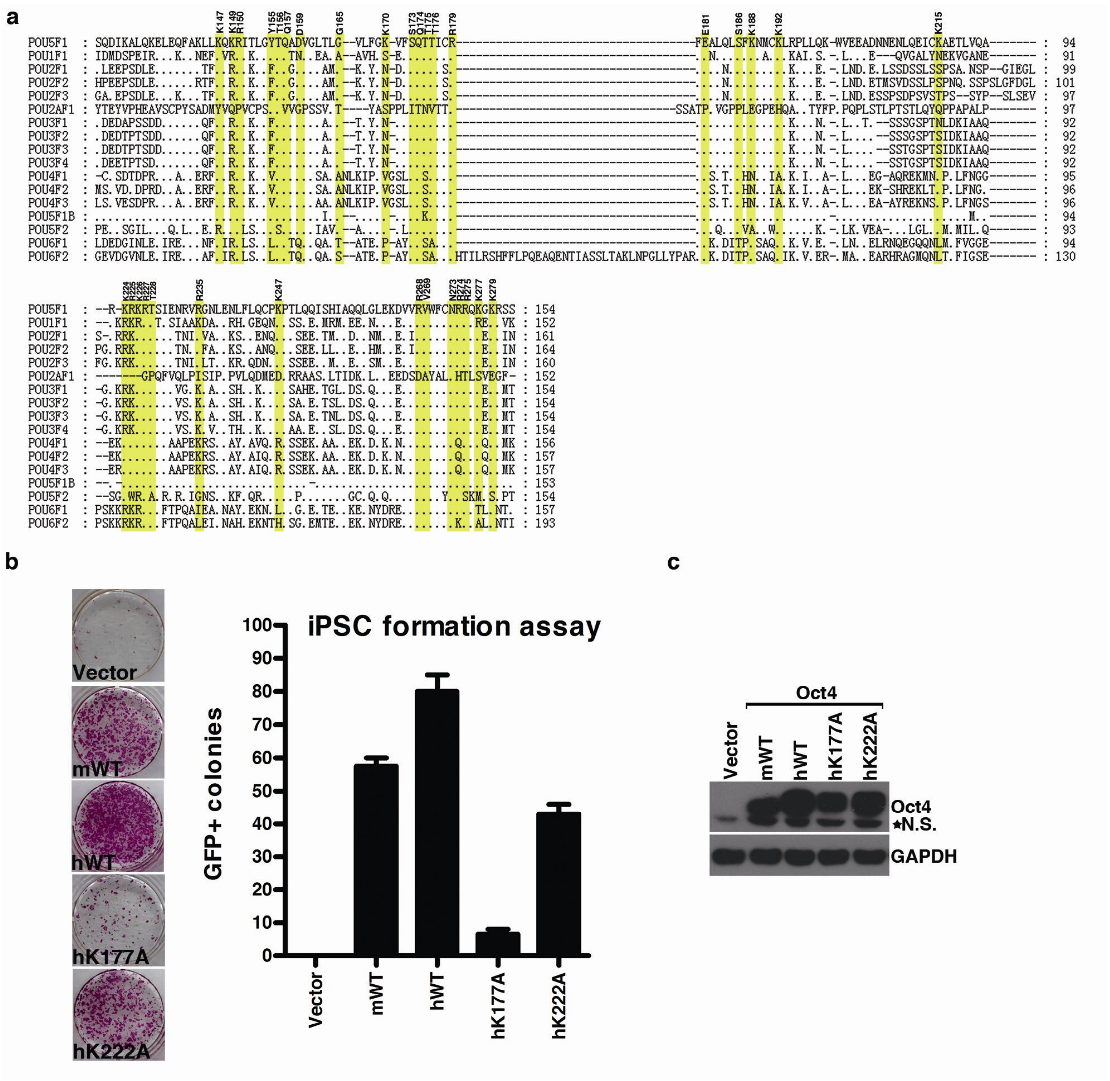

Supplementary Figure S6 Specific residues make human Oct4 unique in reprogramming.

(**a**) Sequence alignment of human POU domain-containing proteins. The numbering at the top is according to mouse Oct4 numbering.

(**b**) AP staining of GFP+ colonies formed by human Oct4 WT, K177A and K222A mutants. Counting of GFP+ colonies is shown at the right. Data are representative of two independent experiments. Statistics analysis was performed using a t-test (mean and s.d. of duplicate assays).

(**c**) Protein stability of hOct4 WT, the K177A and the K222A mutants in MEF cells.

Supplementary Table 1: Smmary of key Oct4 residues											
GFP+ clonies(% of WT		Defective residues information (compared to WT)				Oct4^{POU}:PORE complex crystal structure (3L1P) was analyzed using Pymol software and analysis results are shown below.				putative or reported post translation modification sites	
			Reporter assay							PTM sites	Source
		Protein stability in MEF	6xCR4	5xW	Localization in nucleus		based on POU domain highly conserved between POU family members and available 1HF0 crystal structure, we repaired side chains of K188, K192,	Residues directly interact with DNA(backbone phosphate or base)	Residues directly interacting with functional residues are shown in black which were not tested in this study, and in blue which were already included in our iPSC screening and shown strong defects in reprogramming experiments. Five C to S mutants from crystal		

							**K226, K277 and K279 in 3L1P.**		**structure are shown in red.**		
Vector	0										
WT	100										
S7A	102.5 3										
S12A	84.18									putative phosporylation site	PhosphoSite Plus
S21A	92.41										
R32A	152.9 4										
T33A	95.06										
S36A	92.41										
S50A	112.0 3										
S56A	110.1 3										
Y62A	79.75										
Y70A	94.94										
T87A	106.9 6									reported phosphorylation site	*Proc. Natl. Acad. Sci. U.*

										in human ES cells (human Oct4-T92)	*S. A.* **109**:7162-7168.
R97A	134.57										
S100A	100									reported phosphorylation site in human ES cells (human Oct4-S105)	*Proc. Natl. Acad. Sci. U. S. A.* **109**:7162-7168.
S102A	91.77									reported phosphorylation site in human ES cells (human Oct4-S107)	*Proc. Natl. Acad. Sci. U. S. A.* **109**:7162-7168.
T105A	94.12										
S106-107A	101.9									reported phosphorylation site in human ES cells (human Oct4-S111)	*Proc. Natl. Acad. Sci. U. S. A.* **109**:7162-7168, *J. Biol. Chem.* **287**:38279-38288.
R113A	97.5										
K118A	70.34									reported sumoylation site	*J. Bio. Chem.* **282**:21551-21560; *FASEB J.* **21**:3042-51.
K121A	95.33										
T125A	82.35										
S129A	106.33										

K133A	99.17									putative ubiquitination site	PhosphoSite Plus
K137A	101.67									putative ubiquitination site	PhosphoSite Plus
K144A	127.5							H-bond to backbone phosphate (4.4 Å)		putative ubiquitination site	PhosphoSite Plus
K147A	0	low	low	low	YES				H-bond to A143, I151		
K149A	0	extremely low	low	extremely low	YES				H-bond to L-145, L146, L153		
R150A	0	low	extremely low	extremely low	YES			H-bond to backbone phosphate	H-bond to L146, G154, Y155, E181		
T152A	100										
Y155A	14.36	slightly low	low	sightly low	YES				H-bond to R150, E208, Q211		
T156A	3.08	slightly low	low	low	YES				H-bond to D159, V160	putative phosphorylation site	PhosphoSite Plus
Q157A	12	normal	extremely low	extremely low	YES			H-bond to backbone phospate	H-bond to G161, E181		
D159A	9.33	normal	slightly low	slightly low	YES				H-bond to T156, T163, K215		
T163A	101.67										
G165A	2	normal	slightly low	slightly low	YES				H-bond to L162, G169		
K170A	2.5	normal	slightly low	slightly low	YES	K side chain missing					
S173A	4.1	slightly low	low	extremely low	YES			H-bond to backbone	H-bond to T176, I177	putative phosphorylation site	PhosphoSite Plus

								phospate			
Q174 A	0	normal	extremel y low	normal	YES			H-bond to base	H-bond to S178		
T175A	1.03	normal	slightly low	sightly low	YES			H-bond to base	H-bond to R179		
T176A	2.05	slightly low	low	low	YES			H-bond to backbone phospate	H-bond to S173, R179, F180		
								H-bond to backbone phospate(C178 was not analyzed in this study)			
R179A	0.83	slightly low	extremel y low	extremel y low	YES			H-bond to base	H-bond to T175, T176, L183, Q184		
E181A	0.67	low	extremel y low	extremel y low	YES				H-bond to R150, Q157, I177, S178		
S186A	1.54	extremely low	extremel y low	extremel y low	YES			H-bond to backbone phospate	H-bond to N189, M190		
K188A	30.67	normal	normal	slightly low	YES	K side chain missin g	side chain was repaired		H-bond to K192		
								H-bond to backbone phospate(N189 was not analyzed in this study)			
K192A	13.33	normal	normal	slightly low	YES	K side chain missin g	side chain was repaired	H-bond to backbone phospate	H-bond to K188, N189		
R194A	71.67										

K199A	81.67									putative ubiquitination site	PhosphoSite Plus
K215A	39.33	normal	normal	low	YES	K side chain missing			H-bond to D159, Q211	putative ubiquitination site	PhosphoSite Plus
S216A	112.03										
T218A	147.06										
R223A	96.67										
K224A	39.33	normal	normal	extremely low	YES	K side chain missing					
R225A	8	normal	normal	extremely low	YES	R side chain missing					
K226A	4	normal	normal	low	YES	K side chain missing	side chain was repaired				
R227A	0.67	normal	normal	extremely low	YES			H-bond to base			
T228A	18.97	normal	normal	sightly low	YES			H-bond to backbone phospate		reported phosphorylation site in human ES and EC cells (human Oct4-T235) and reported glycosylation site	*Proc. Natl. Acad. Sci. U. S. A.* **109**:7162-7168, *Mol. Cell* **48**:1-14, *Cell Stem Cell* **11**:62-74.
S229A	88.61									reported	*Proc. Natl.*

										phosphorylation site in human ES cells (human Oct4-S236)	*Acad. Sci. U. S. A.* **109**:7162-7168.
R233A	107.33										
R235A	11.33	normal	normal	low	YES				H-bond to E239, I230		
S237A	102.53										
T240A	94.12										
K244A	89.33										
K247A	28.67	normal	normal	low	YES			H-bond to backbone phospate			
S249A	88.26										
T254A	82.35										
K264A	93.33										
R268A	24	normal	normal	low	YES			H-bond to backbone phospate	H-bond to K264, D265, S272		
V269A	2	normal	normal	sightly low	YES			non polar contacts with base	H-bond to D265, S272, N273		
C272A	104	normal	normal	normal	YES						
N273A	0	normal	normal	extremely low	YES			H-bond to base	H-bond to V269, Q276, K277		
R274A	0	normal	normal	low	YES				H-bond to E239, W270, G278		
R275A	0	normal	normal	low	YES			H-bond to backbone phospate	H-bond to P246, F271, S272, K279		
								H-bond to base (Q276 was not			

								analyzed in this study)		
K277A	1.33	normal	normal	low	YES	K side chain missin g	side chain was repaired	H-bond to backbone phospate	H-bond to N273		
K279A	8	normal	normal	low	YES	K side chain missin g	side chain was repaired	H-bond to backbone phospate	H-bond to R275		
R280A	83.54										
S281-282A	96.2									reported phosphorylation site in human ES cells (human Oct4-S288)	*Proc. Natl. Acad. Sci. U. S. A.* **109**:7162-7168.
Y285A	96.2										
S286A	89.87										
R288A	109.3 3										
Y291A	91.18										
T294-296A	91.18										
S304A	88.89										
T315A	87.97										
Y318A	96.84										
S320A	98.77										
T324-325A	85.29										
Y327A	41.14	normal	normal	sightly low	YES					putative phosphorylation site	*Plos One* **4**:e4467.
S328A	99.37										
S339A	85.19										

T343A	101.9										
S347-351A	92.5									reported phosphorylation site in human ES cells (human Oct4-S355 and human Oct4-S359)	*Proc. Natl. Acad. Sci. U. S. A.* **109**:7162-7168.

Supplementary Table 2: Vectors, Primers used for library construction

Entry clone	mutagenesis primer pairs		LR reaction with pMXs-CHR-ccdB (destination vector)
	Sense primer	Antisense primer	
pDONR223-mOct4-WT			pMXs-mOct4-WT
pDONR223-mOct4-S7A	GGACACCTGGCTGCAGACTTCGCCTTCTCAC	GTGAGAAGGCGAAGTCTGCAGCCAGGTGTCC	pMXs-mOct4-S7A
pDONR223-mOct4-S12A	GACTTCGCCTTCGCACCCCCACCAGGTG	CACCTGGTGGGGGTGCGAAGGCGAAGTC	pMXs-mOct4-S12A
pDONR223-mOct4-S21A	GGGGGTGATGGGGCAGCAGGGCTGGAG	CTCCAGCCCTGCTGCCCCATCACCCCC	pMXs-mOct4-S21A
pDONR223-mOct4-S36A	CGAACCTGGCTAGCCTTCCAAGGGCCTCC	GGAGGCCCTTGGAAGGCTAGCCAGGTTCG	pMXs-mOct4-S36A
pDONR223-mOct4-S50A	GGAATCGGACCAGGCGCAGAGGTATTGGGGATC	GATCCCCAATACCTCTGCGCCTGGTCCGATTCC	pMXs-mOct4-S50A
pDONR223-mOct4-S56A	GTATTGGGGATCGCCCCATGTCCGCCC	GGGCGGACATGGGGCGATCCCCAATAC	pMXs-mOct4-S56A
pDONR223-mOct4-S100A	GGAGCACGAGTGGAAGCGAATTCAGAGGGAACC	GGTTCCCTCTGAATTCGCTTCCACTCGTGCTCC	pMXs-mOct4-S100A
pDONR223-mOct4-S102A	CGAGTGGAAAGCAACGCAGAGGGAACCTCCTC	GAGGAGGTTCCCTCTGCGTTGCTTTCCACTCG	pMXs-mOct4-S102A
pDONR223-mOct4-S106-107A	CTCAGAGGGAACCGCCGCTGAGCCCTGTGCCGAC	GTCGGCACAGGGCTCAGCGGCGGTTCCCTCTGAG	pMXs-mOct4-S106-107A
pDONR223-mOct4-S129A	CCAACTCCCGAGGAGGCCCAGGACATGAAAGCC	GGCTTTCATGTCCTGGGCCTCCTCGGGAGTTGG	pMXs-mOct4-S129A
pDONR223-mOct4-S173A	GGAAAGGTGTTCGCCCAGACCACCATCTGTCG	CGACAGATGGTGGTCTGGGCGAACACCTTTCC	pMXs-mOct4-S173A
pDONR223-mOct4-S186A	GAGGCCTTGCAGCTCGCCCTTAAGAACATGTG	CACATGTTCTTAAGGGCGAGCTGCAAGGCCTC	pMXs-mOct4-S186A
pDONR223-mOct4-S216A	CAGGAGATATGCAAAGCGGAGACCCTGGTGCAGG	CCTGCACCAGGGTCTCCGCTTTGCATATCTCCTG	pMXs-mOct4-S216A
pDONR223-mOct4-S229A	GAGAAAGCGAACTGCCATTGAGAACCGTGTGAGG	CCTCACACGGTTCTCAATGGCAGTTCGCTTTCTC	pMXs-mOct4-S229A
pDONR223-mOct4-S237A	CCGTGTGAGGTGGGCTCTGGAGACCATGTTTC	GAAACATGGTCTCCAGAGCCCACCTCACACGG	pMXs-mOct4-S237A
pDONR223-mOct4-S249A	GTGCCCGAAGCCCGCCCTACAGCAGATC	GATCTGCTGTAGGGCGGGCTTCGGGCAC	pMXs-mOct4-S249A
pDONR223-mOct4-S281-282A	CCAGAAGGGCAAAAGAGCAGCTATTGAGTATTCCCAAC	GTTGGGAATACTCAATAGCTGCTCTTTTGCCCTTCTGG	pMXs-mOct4-S281-282A
pDONR223-mOct4-S286A	GATCAAGTATTGAGTATGCCCAACGAGAAGAG	CTCTTCTCGTTGGGCATACTCAATACTTGATC	pMXs-mOct4-S286A

pDONR223-mOct4-S304A	GGGGGGGCTGTAGCCTTTCCTCTGCCCCC	GGGGGCAGAGGAAAGGCTACAGCCCCCCC	pMXs-mOct4-S304A
pDONR223-mOct4-S320A	CCAGGCTATGGAGCCCCCCACTTCACCACAC	GTGTGGTGAAGTGGGGGGCTCCATAGCCTGG	pMXS-mOct4-S320A
pDONR223-mOct4-S328A	CACCACACTCTACGCAGTCCCTTTTCCTGAGGGC	GCCCTCAGGAAAAGGGACTGCGTAGAGTGTGGTG	pMXs-mOct4-S328A
pDONR223-mOct4-S339A	GAGGCCTTTCCCGCTGTTCCCGTCACTGCTC	GAGCAGTGACGGGAACAGCGGGAAAGGCCTC	pMXS-mOct4-S339A
pDONR223-mOct4-S347-351A	CACTGCTCTGGGCGCTCCCATGCATGCAAACGA CTACAAAG	CTTTGTAGTCGTTTGCATGCATGGGAGCGCCCAG AGCAGTG	pMXS-mOct4-S347-351A
pDONR223-mOct4-T33A	CTGGGTGGATTCTCGAGCCTGGCTAAGCTTCC	GGAAGCTTAGCCAGGCTCGAGAATCCACCCAG	pMXS-mOct4-T33A
pDONR223-mOct4-T87A	GTTGGCGTGGAGGCTTTGCAGCCTGAGGGC	GCCCTCAGGCTGCAAAGCCTCCACGCCAAC	pMXS-mOct4-T87A
pDONR223-mOct4-T105A	GCAACTCAGAGGGAGCCTCCTCTGAGCCCTG	CAGGGCTCAGAGGAGGCTCCCTCTGAGTTGC	pMXS-mOct4-T105A
pDONR223-mOct4-T125A	GAGAAGGTGGAACCAGCTCCCGAGGAGTCCC	GGGACTCCTCGGGAGCTGGTTCCACCTTCTC	pMXS-mOct4-T125A
pDONR223-mOct4-T152A	GCAGAAGAGGATCGCCTTGGGGTACACCCAG	CTGGGTGTACCCCAAGGCGATCCTCTTCTGC	pMXs-mOct4-T152A
pDONR223-mOct4-T156A	CACCTTGGGGTACGCCCAGGCCGACGTG	CACGTCGGCCTGGGCGTACCCCAAGGTG	pMXs-mOct4-T156A
pDONR223-mOct4-T163A	GACGTGGGGCTCGCCCTGGGCGTTCTC	GAGAACGCCCAGGGCGAGCCCCACGTC	pMXs-mOct4-T163A
pDONR223-mOct4-T175A	GGTGTTCAGCCAGGCCACCATCTGTCGCTTCGA GG	CCTCGAAGCGACAGATGGTGGCCTGGCTGAACAC C	pMXs-mOct4-T175A
pDONR223-mOct4-T176A	GGTGTTCAGCCAGACCGCCATCTGTCGCTTCGA GG	CCTCGAAGCGACAGATGGCGGTCTGGCTGAACAC C	pMXs-mOct4-T176A
pDONR223-mOct4-T218A	GATATGCAAATCGGAGGCCCTGGTGCAGGCC	GGCCTGCACCAGGGCCTCCGATTTGCATATC	pMXs-mOct4-T218A
pDONR223-mOct4-T228A	CGGAAGAGAAAGCGAGCTAGCATTGAGAACCGT G	CACGGTTCTCAATGCTAGCTCGCTTTCTCTTCCG	pMXs-mOct4-T228A
pDONR223-mOct4-T240A	GGTGGAGTCTGGAGGCCATGTTTCTGAAGTGCC	GGCACTTCAGAAACATGGCCTCCAGACTCCACC	pMXs-mOct4-T240A
pDONR223-mOct4-T254A	CCCTACAGCAGATCGCTCACATCGCCAATCAG	CTGATTGGCGATGTGAGCGATCTGCTGTAGGG	pMXS-mOct4-T254A
pDONR223-mOct4-T294-296A	GAAGAGTATGAGGCTGCAGGGGCACCTTTCCCA GGGGGG	CCCCCCTGGGAAAGGTGCCCCTGCAGCCTCATAC TCTTC	pMXS-mOct4-T294-296A
pDONR223-mOct4-T315A	CCCCACTTTGGCGCCCCAGGCTATGGAAG	CTTCCATAGCCTGGGGCGCCAAAGTGGGG	pMXs-mOct4-T315A
pDONR223-mOct4-T324-325A	GGAAGCCCCCACTTCGCCGCACTCTACTCAGTC CCTTTTCC	GGAAAAGGGACTGAGTAGAGTGCGGCGAAGTGG GGGCTTCC	pMXs-mOct4-T324-325A
pDONR223-mOct4-T343A	CTCTGTTCCCGTCGCTGCTCTGGGCTCTC	GAGAGCCCAGAGCAGCGACGGGAACAGAG	pMXs-mOct4-T343A
pDONR223-mOct4-Y62A	CCATGTCCGCCCGCAGCCGAGTTCTGCGGAG	CTCCGCAGAACTCGGCTGCGGGCGGACATGG	pMXs-mOct4-Y62A
pDONR223-mOct4-Y70A	GGAGGGATGGCAGCCTGTGGACCTCAGG	CCTGAGGTCCACAGGCTGCCATCCCTCC	pMXs-mOct4-Y70A
pDONR223-mOct4-Y155A	GGATCACCTTGGGGGCCACCCAGGCCGACGTG	CACGTCGGCCTGGGTGGCCCCCAAGGTGATCC	pMXs-mOct4-Y155A
pDONR223-mOct4-Y285A	GCAAAAGATCAAGTATTGAGGCTTCCCAACGAGA AGAGTATG	CATACTCTTCTCGTTGGGAAGCCTCAATACTTGAT CTTTTGC	pMXs-mOct4-Y285A

pDONR223-mOct4-Y291A	CCCAACGAGAAGAGGCTGAGGCTACAGGGACAC	GTGTCCCTGTAGCCTCAGCCTCTTCTCGTTGGG	pMXs-mOct4-Y291A
pDONR223-mOct4-Y318A	GGCACCCCAGGCGCTGGAAGCCCCCAC	GTGGGGGCTTCCAGCGCCTGGGGTGCC	pMXs-mOct4-Y318A
pDONR223-mOct4-Y327A	CCACTTCACCACACTCGCCTCAGTCCCTTTTCC	GGAAAAGGGACTGAGGCGAGTGTGGTGAAGTGG	pMXs-mOct4-Y327A
pDONR223-mOct4-K118A	CCCAATGCCGTGGCGTTGGAGAAGGTGGAACCA ACTCC	GGAGTTGGTTCCACCTTCTCCAACGCCACGGCATT GGG	pMXs-mOct4-K118A
pDONR223-mOct4-K121A	GCCGTGAAGTTGGAGGCGGTGGAACCAACTCCC	GGGAGTTGGTTCCACCGCCTCCAACTTCACGGC	pMXs-mOct4-K121A
pDONR223-mOct4-K133A	GTCCCAGGACATGGCAGCCCTGCAGAAGGAG	CTCCTTCTGCAGGGCTGCCATGTCCTGGGAC	pMXs-mOct4-K133A
pDONR223-mOct4-K137A	CATGAAAGCCCTGCAGGCGGAGCTAGAACAG	CTGTTCTAGCTCCGCCTGCAGGGCTTTCATG	pMXs-mOct4-K137A
pDONR223-mOct4-K144A	CTAGAACAGTTTGCCGCGCTGCTGAAGCAGAAG	CTTCTGCTTCAGCAGCGCGGCAAACTGTTCTAG	pMXs-mOct4-K144A
pDONR223-mOct4-K147A	GCCAAGCTGCTGGCGCAGAAGAGGATCACC	GGTGATCCTCTTCTGCGCCAGCAGCTTGGC	pMXs-mOct4-K147A
pDONR223-mOct4-K149A	GCTGCTGAAGCAGGCGAGGATCACCTTGGGG	CCCCAAGGTGATCCTCGCCTGCTTCAGCAGC	pMXs-mOct4-K149A
pDONR223-mOct4-K170A	GGCGTTCTCTTTGGAGCGGTGTTCAGCCAG	CTGGCTGAACACCGCTCCAAAGAGAACGCC	pMXs-mOct4-K170A
pDONR223-mOct4-K188A	GCAGCTCAGCCTTGCGAACATGTGTAAGCTGC	GCAGCTTACACATGTTCGCAAGGCTGAGCTGC	pMXs-mOct4-K188A
pDONR223-mOct4-K192A	CTTAAGAACATGTGTGCGCTGCGGCCCCTG	CAGGGGCCGCAGCGCACACATGTTCTTAAG	pMXs-mOct4-K192A
pDONR223-mOct4-K199A	CCCCTGCTGGAGGCGTGGGTGGAGGAAG	CTTCCTCCACCCACGCCTCCAGCAGGGG	pMXs-mOct4-K199A
pDONR223-mOct4-K215A	CAGGAGATATGCGCATCGGAGACCCTGGTG	CACCAGGGTCTCCGATGCGCATATCTCCTG	pMXs-mOct4-K215A
pDONR223-mOct4-K224A	GTGCAGGCCCGGGCGAGAAAGCGAACTAGC	GCTAGTTCGCTTTCTCGCCCGGGCCTGCAC	pMXs-mOct4-K224A
pDONR223-mOct4-K226A	GCCCGGAAGAGAGCGCGAACTAGCATTGAG	CTCAATGCTAGTTCGCGCTCTCTTCCGGGC	pMXs-mOct4-K226A
pDONR223-mOct4-K244A	GAGACCATGTTTCTGGCGTGCCCGAAGCCCTCC	GGAGGGCTTCGGGCACGCCAGAAACATGGTCTC	pMXS-mOct4-K244A
pDONR223-mOct4-K247A	CTGAAGTGCCCGGCGCCCTCCCTACAG	CTGTAGGGAGGGCGCCGGGCACTTCAG	pMXs-mOct4-K247A
pDONR223-mOct4-K264A	CAGCTTGGGCTAGAGGCGGATGTGGTTCGAG	CTCGAACCACATCCGCCTCTAGCCCAAGCTG	pMXs-mOct4-K264A
pDONR223-mOct4-K277A	GTAACCGGCGCCAGGCGGGCAAAAGATCAAG	CTTGATCTTTTGCCCGCCTGGCGCCGGTTAC	pMXS-mOct4-K277A
pDONR223-mOct4-K279A	CGCCAGAAGGGCGCAAGATCAAGTATTG	CAATACTTGATCTTGCGCCCTTCTGGCG	pMXS-mOct4-K279A
pDONR223-mOct4-R32A	GGCTGGGTGGATTCTGCAACCTGGCTAAGC	GCTTAGCCAGGTTGCAGAATCCACCCAGCC	pMXs-mOct4-R32A
pDONR223-mOct4-R97A	CAGGCAGGAGCAGCAGTGGAAAGCAACTCAG	CTGAGTTGCTTTCCACTGCTGCTCCTGCCTG	pMXS-mOct4-R97A
pDONR223-mOct4-R113A	CCCTGTGCCGACGCCCCCAATGCCGTG	CACGGCATTGGGGGCGTCGGCACAGGG	pMXs-mOct4-R113A
pDONR223-mOct4-R150A	CTGCTGAAGCAGAAGGCGATCACCTTGGGG	CCCCAAGGTGATCGCCTTCTGCTTCAGCAG	pMXS-mOct4-R150A
pDONR223-mOct4-R179A	CAGACCACCATCTGTGCCTTCGAGGCCTTGCAG	CTGCAAGGCCTCGAAGGCACAGATGGTGGTCTG	pMXS-mOct4-R179A
pDONR223-mOct4-R194A	CATGTGTAAGCTGGCGCCCCTGCTGGAGAAG	CTTCTCCAGCAGGGGCGCCAGCTTACACATG	pMXs-mOct4-R194A
pDONR223-mOct4-R223A	CTGGTGCAGGCCGCGAAGAGAAAGCGAAC	GTTCGCTTTCTCTTCGCGGCCTGCACCAG	pMXS-mOct4-R223A
pDONR223-mOct4-R225A	CAGGCCCGGAAGGCAAAGCGAACTAGCATTGAG	CTCAATGCTAGTTCGCTTTGCCTTCCGGGCCTG	pMXs-mOct4-R225A
pDONR223-mOct4-R227A	GCCCGGAAGAGAAAGGCAACTAGCATTGAGAAC	CGGTTCTCAATGCTAGTTGCCTTTCTCTTCCGGGC	pMXs-mOct4-R227A

	CG		
pDONR223-mOct4-R233A	CTAGCATTGAGAACGCTGTGAGGTGGAGTCTG	CAGACTCCACCTCACAGCGTTCTCAATGCTAG	pMXs-mOct4-R233A
pDONR223-mOct4-R235A	GAGAACCGTGTGGCGTGGAGTCTGGAG	CTCCAGACTCCACGCCACACGGTTCTC	pMXs-mOct4-R235A
pDONR223-mOct4-R268A	GGGCTAGAGAAGGATGTGGTTGCAGTATGGTTC TGTAAC	GTTACAGAACCATACTGCAACCACATCCTTCTCTA GCCC	pMXs-mOct4-R268A
pDONR223-mOct4-R274A	GTATGGTTCTGTAACGCGCGCCAGAAGGGC	GCCCTTCTGGCGCGCGTTACAGAACCATAC	pMXs-mOct4-R274A
pDONR223-mOct4-R275A	GGTTCTGTAACCGGGCCCAGAAGGGCAAAAGAT C	GATCTTTTGCCCTTCTGGGCCCGGTTACAGAACC	pMXs-mOct4-R275A
pDONR223-mOct4-R280A	CGCCAGAAGGGCAAAGCATCAAGTATTGAGTATT CCC	GGGAATACTCAATACTTGATGCTTTGCCCTTCTGG CG	pMXs-mOct4-R280A
pDONR223-mOct4-R288A	CAAGTATTGAGTATTCCCAAGCAGAAGAGTATGA GGCTACAGGG	CCCTGTAGCCTCATACTCTTCTGCTTGGGAATACT CAATACTTG	pMXs-mOct4-R288A
pDONR223-mOct4-C272A	GTTCGAGTATGGTTCGCTAACCGGCGCCAG	CTGGCGCCGGTTAGCGAACCATACTCGAAC	pMXs-mOct4-C272A
pDONR223-mOct4-D159A	GGGTACACCCAGGCCGCCGTGGGGCTCACCCT G	CAGGGTGAGCCCCACGGCGGCCTGGGTGTACCC	pMXs-mOct4-D159A
pDONR223-mOct4-E181A	CCATCTGTCGCTTCGCGGCCTTGCAGCTCAGC	GCTGAGCTGCAAGGCCGCGAAGCGACAGATGG	pMXs-mOct4-E181A
pDONR223-mOct4-G165A	GTGGGGCTCACCCTGGCCGTTCTCTTTGGAAAG G	CCTTTCCAAAGAGAACGGCCAGGGTGAGCCCCAC	pMXs-mOct4-G165A
pDONR223-mOct4-N273A	CGAGTATGGTTCTGTGCCCGGCGCCAGAAGG	CCTTCTGGCGCCGGGCACAGAACCATACTCG	pMXs-mOct4-N273A
pDONR223-mOct4-Q157A	CCTTGGGGTACACCGCGGCCGACGTGGGGC	GCCCCACGTCGGCCGCGGTGTACCCCAAGG	pMXs-mOct4-Q157A
pDONR223-mOct4-Q174A	GGAAAGGTGTTCAGCGCGACCACCATCTGTCGC	GCGACAGATGGTGGTCGCGCTGAACACCTTTCC	pMXs-mOct4-Q174A
pDONR223-mOct4-V269A	GGATGTGGTTCGAGCATGGTTCTGTAACCGG	CCGGTTACAGAACCATGCTCGAACCACATCC	pMXs-mOct4-V269A
pDONR223-mOct4-S186D	GAGGCCTTGCAGCTCGACCTTAAGAACATGTG	CACATGTTCTTAAGGTCGAGCTGCAAGGCCTC	pMXs-mOct4-S186D
pDONR223-mOct4-S186E	GAGGCCTTGCAGCTCGAACTTAAGAACATGTG	CACATGTTCTTAAGTTCGAGCTGCAAGGCCTC	pMXs-mOct4-S186E
**Other constructs**	**Primer pairs**		**Source**
	**Sense primer**	**Antisense primer**	
pDONR223-mOct4	CGGTGTACAAAAAAGCAGGCTGGATGGCTGGAC ACCTGGCTTCAGACTTCG	CGGTGTACAAGAAAGCTGGGTTTCACTTGTCATCG TCGTCTTTGTAGTCGTTTGAATGCATGGGAGAGCC CAGAGC	
pMXs-CHR-ccdB	CGGTTAATTAA ATAAGCAGAGCTCTCTGGCTAACTG	CGGCTCGAGCATTACTACGTAGAATCGAGACCGA	

pUAST-SapI-1xCR4	CGGGGTACC GCTCTTCGAACTGGGTGTGGGGAGGTTGTAGC	CGGTCTAGA GCTCTTCGGTTTAGCAGATTAAGGAAGGGC	
pUAST-NheI-1xCR4	CGGGGTACC GCTCTTCGAACTGGGTGTGGGGAGGTTGTAGC	CGGTCTAGAGCTAGCGGTTTAGCAGATTAAGGAAGGGC	
pUAST-6xCR4			subcloned from pUAST-SapI-6XCR4
pGL4.2-6xCR4			subcloned from pUAST-6XCR4
pGL4.2-5xW	CCCCTGAGCAAAACACCACCTGGGTAATTTGCATTTCTAAAATAAGTCGA	GGGTCGACTTATTTTAGAAATGCAAATTACCCAGGTGGTGTTTTGCTCAG	
pGL4.2-5xW-37TK	CGGCTCGAGGGTCCACTTCGCATATTAAGGTGACG	CGGAGATCTCGGCACGCTGTTGACGCTGTTAAGCGGGTCGCTGC	
pMXs-mOct4-△NTD-Flag	CGGGGATCCATGGACATGAAAGCCCTGCAGAAGG	CGGGCGGCCGCTCACTTGTCGTCATCGTCTTTGTAGTCGTTTGAATGCATGGG	
pMXs-mOct4-△CTD-Flag	CGGGGATCCATGGCTGGACACCTGGCTTCAGAC	CGGGCGGCCGCTCACTTGTCGTCATCGTCTTTGTAGTCACTTGATCTTTTGCC	
pMXs-mOct4-△POU(NTD+CTD)-Flag			
pMXs-mOct4-NTD	CGGGGATCCATGGCTGGACACCTGGCTTCAGAC	CGGGAATTCCTGGGACTCCTCGGGAGTTGGTTCC	
pMXs-mOct4-CTD-Flag	CGGGAATTCATTGAGTATTCCCAACGAGAAGAG	CGGGCGGCCGCTCACTTGTCGTCATCGTCTTTGTAGTCGTTTGAATGCATGGG	
pMXs-mOct4-POU-Flag	CGGGGATCCATGGACATGAAAGCCCTGCAGAAGG	CGGGCGGCCGCTCACTTGTCGTCATCGTCTTTGTAGTCACTTGATCTTTTGCC	
pMXs-mOct4-△NTD-YAP^{TAD}-Flag	CGGGGATCCATGGACATGAAAGCCCTGCAGAAGG	CGGGCGGCCGCGTTTGAATGCATGGGAGAGCCCAGAG	
pMXs-mOct4-△CTD-YAP^{TAD}-Flag	CGGGGATCCATGGCTGGACACCTGGCTTCAGAC	CGGGCGGCCGCACTTGATCTTTTGCCCTTCTGGCGC	
pMXs-mOct4-△POU-YAP^{TAD}-Flag	CGGGGATCCATGGCTGGACACCTGGCTTCAGAC	CGGGCGGCCGCGTTTGAATGCATGGGAGAGCCCAGAG	
pMXs-mOct4-POU-YAP^{TAD}-Flag	CGGGGATCCATGGACATGAAAGCCCTGCAGAAGG	CGGGCGGCCGCACTTGATCTTTTGCCCTTCTGGCGC	
pMXs-YAP^{TAD}-Flag	CGGTTAATTAAATGCAGGGAGGCGTCCTGGGTGGAG	CGGGGATCCCTACTTGTCATCGTCATCCTTGTAATCTAACC	

pMXs-EGFP-FLAG	CGGTTAATTAAATGGTGAGCAAGGGCGAGGAGC TGTTC	CGGCTCGAGTCACTTGTCATCGTCGTCTTTGTAGT CCTTGTACAGCTCGTCCATGCCGAGAGTG	
pMXs-mSox2-Myc	CGGGAATTCATGTATAACATGATGGAGACGGAG CTGAAGC	CGGGCGGCCGCTCACAGATCCTCTTCTGAGATGA GTTTTTGTTCCATGTGCGACAGGGG	
pMXs-mOct4-WT-YAP^{TAD}-Flag			*Stem Cell Reports* **2**:1-9.
pMXS-mKlf4			purchased from addgene
pMXs-mNanog			purchased from addgene
pMXs-m-cMyc			purchased from addgene
pMXs-hOct4-WT-Flag	CGGGGATCCATGGCGGGACACCTGGCTTCGGAT TTCG	CGGGCGGCCGCTCACTTGTCGTCATCGTCTTTGT AGTCGTTTGAATGCATGGGAGAG	
pMXs-hOct4-K177A-Flag	GGTTCTATTTGGGGCGGTATTCAGCCAAAC	GTTTGGCTGAATACCGCCCCAAATAGAACC	
pMXs-hOct4-K222A-Flag	CAGGAGATATGCGCAGCAGAAACCCTC	GAGGGTTTCTGCTGCGCATATCTCCTG	
